# Integrated Analysis of Transcriptomic and Proteomic Data

**DOI:** 10.2174/1389202911314020003

**Published:** 2013-04

**Authors:** Saad Haider, Ranadip Pal

**Affiliations:** Department of Electrical and Computer Engineering, Texas Tech University, Lubbock, TX, 79409, USA

**Keywords:** Integrated omics, Data fusion approaches, Transcriptome, Proteome, Joint modeling, Combined analysis review.

## Abstract

Until recently, understanding the regulatory behavior of cells has been pursued through independent analysis of the transcriptome or the proteome. Based on the central dogma, it was generally assumed that there exist a direct correspondence between mRNA transcripts and generated protein expressions. However, recent studies have shown that the correlation between mRNA and Protein expressions can be low due to various factors such as different half lives and post transcription machinery. Thus, a joint analysis of the transcriptomic and proteomic data can provide useful insights that may not be deciphered from individual analysis of mRNA or protein expressions. This article reviews the existing major approaches for joint analysis of transcriptomic and proteomic data. We categorize the different approaches into eight main categories based on the initial algorithm and final analysis goal. We further present analogies with other domains and discuss the existing research problems in this area.

## 1 INTRODUCTION

 One of the significant objectives of Systems Biology is to understand the regulation of cell behavior through interactions of various components in the regulome (regulation components in the cell such as mRNA, proteins, metabolites etc.). Two important observational categories involves (a) measurement of transcriptomic profiles through techniques such as microarray, RNA-seq etc. and (b) measurement of proteomic profiles through techniques such as gel electrophoresis and mass spectrometry. Some of the data measurement techniques may involve destruction of the living cell and thus joint measurement of both transcripts and proteins in a single cell will not be feasible by such methods. Furthermore, some approaches may provide expression data on the average behavior of a collection of cells and not the expression distribution of the cells. Thus, understanding the limitations and assumptions in the data measurement techniques used for measuring the transcriptomic and proteomic profiles is essential before conducting a joint analysis of the two data sources. Section 2 of this article provides a review of the various approaches used for measuring transcriptomic and proteomic profiles. 

 The next step in developing a joint model of the two domains involves comprehending the differences in the expression of the mRNAs and proteins. Studies [1-5] have shown that there can be poor correlation between mRNA and protein expression data from same cells under similar conditions. Section 3 of this article discusses and provides possible reasons for the lack of correlation between mRNA and Protein expressions.

 Finally, the type of extracted transcriptomic and proteomic data and the ultimate goal of analysis will dictate the manner of the joint analysis of the two domains. Section 4 discusses the various approaches for the integrated analysis of transcriptomic and proteomic profiles. We divide the various available approaches into eight main categories and provide an initial overview of the techniques followed by specific examples in section 5. 

 Section 6 provides analogies between biological scenarios and other physical scenarios so that approaches used for the analysis of one can throw insights and be possibly used for the analysis of the other. We compare the gene-transcriptome-proteome network with an organizational command structure and large scale social network.

 Section 7 provides conclusions and future research directions. 

 The current review focuses on uncovering the primary categories of approaches that have been proposed for fusion of transcriptomic and proteomic data. In comparison, existing reviews on joint transcriptomic and proteomic profiling focuses on specific aspects of combined analysis. For instance, Catherine Hack [6] focuses on different statistical methods for correlation between transcriptomic and proteomic datasets. Cox *et al.* [7] reviews different methods for comparison of microarray and proteomic datasets along with clustering and merging options for these datasets. Nie *et al.* [8] focuses on attempts to develop various statistical tools for improving the chances of capturing a relationship between transcriptomic and proteomic data along with different transformation and normalization techniques for data, effects on measurement errors and challenges of missing values in datasets. A significant part of the paper by Hecker *et al.* [9] reviews approaches to build dynamic models of transcriptomic and/or proteomic network. Simon Rogers [10] described the available statistical tools for bridging multi-omics data.

## 2 TECHNIQUES FOR TRANSCRIPTOMIC AND PROTEOMIC DATASET GENERATION

### 2.1 Methods for Transcriptomic Profiling

 Current transcriptomic profiling techniques include DNA microarray, cDNA amplified fragment length polymorphism (cDNA-AFLP), expressed sequence tag (EST) sequencing, serial analysis of gene expression (SAGE), massive parallel signature sequencing (MPSS), RNA-seq etc.

 Among the above mentioned technologies, DNA microarray [11] is the most widely used one. But, its application is dependent on the availability of complete genome sequence or knowledge of significant amount of transcript sequence. This technique has evolved from Southern blotting [12] and has been widely accepted as an inexpensive analog technique for high-throughput transcriptomic profiling. cDNA-AFLP [13] is a highly sensitive method which allows the detection of low-abundance mRNAs. Recent examples of cDNA-AFLP based transcriptomic studies are documented in [14] and [15]. EST ^1^ sequencing is another approach for transcriptomic profiling which has been used in a large number of transcriptomic studies (e.g. [16, 17]). SAGE [18] is a RNA-sequencing based transcriptomic profiling method that can be used to analyze large number of transcripts quantitatively and simultaneously (e.g. [19] and [3]). MPSS [20] is another sequenced based approach for profiling transcriptomic data which is somewhat similar to SAGE but with a substantial difference in sequencing approach and with different approach to biochemical manipulation (e.g. [21] and [22]).

 The most recent technology for transcriptomic profiling is RNA-Seq [23] which is considered as a revolutionary tool for this purpose. Eukaryotic transcriptomic profiles are primarily analyzed with this technique and it has been already applied for transcriptomic analysis of several organisms including *Saccharomyces cerevisiae*, *Schizosaccharomyces pombe*, *Arabidopsis thaliana*, mouse and human cells [24-29].

 RNA-Seq technology shows clear advantages over existing profiling technologies in terms of amount of sequence coverage, revealing new transcriptomic insights, accuracy of defining transcription level, etc. However, existing microarray technology still remains reliable to many researchers for various reasons (explained in an article by Nathan Blow [30]). Overall comparison of existing technologies and most recent RNA-Seq technology can be found in recent reviews by Nicole Roy *et al.* [31] and Schirmer *et al.* [32]. Use of different transcriptomic technologies and their success on Amyotrophic Lateral Sclerosis study was discussed in recent review [33]. Also, the omics-era technologies for systems-level understanding of Streptomyceshas have been discussed in a recent review [34]. Genome-wide copy number analysis [35] is another area where extensive use of different transcriptomic technologies is exercised.

### 2.2 Methods for Proteomic Profiling

 Current *state of the art* proteomic technologies include: 2-dimensional difference gel electrophoresis (2D DIGE), matrix-assisted laser desorption/ionization (MALDI) imaging mass spectrometry, electron transfer dissociation mass spectrometry and reverse-phase protein array.

 2D-DIGE is a form of gel-electrophoresis which can label 3 different samples of proteins with fluorescent dyes. This method overcomes the limitations due to inter-gel variation in traditional 2D gel electrophoresis technique (2D-GE) [36] of proteomic profiling. Despite the limitation in 2D-GE method, it is still a mature proteomic profiling technique backed by 3 decades of research. Examples of proteomic study using 2D-GE can be found in [37] and [38]; whereas [39] and [40] provide examples of using 2D-DIGE technique in proteomic study. A detailed comparison between these 2 techniques can be found in the article by Marouga *et al.* [41]. MALDI imaging mass spectrometry [42] is a unique technique for identification of biomarkers in different diseases. Studies of proteomics profiling using this technique include [43] and [44]. Mass spectrometry based quantitative proteomic analysis is another form of proteomic profiling which is followed by 2D-GE. Here, intensity of protein stain is measured to find the existence and amount of protein present in a sample. Liquid chromatography mass spectrometry (LC-MS) (example studies [45] and [46]), liquid chromatography-tandem mass spectrometry (LC-MS/MS) (example studies [47] and [48]), in-gel tryptic digestion followed by liquid chromatography-tandem mass spectrometry (geLC-MS/MS) (example studies [49] and [50]) are different versions of mass spectrometry techniques used in proteomic profiling. Electron transfer dissociation (ETD) mass spectrometry [51] is another form of proteomic study which is a method of fragmenting ions in a mass spectrometer. [52] and [53] are examples of proteomic studies that use ETD. *Reverse-phase protein array* [54] is a protein microarray technology that has use in quantitative analysis of protein expressions in various kinds of cells including cancer cells, body fluids and tissues (example studies [55] and [56]). Use of several technologies stated above on prognosis and outcome of the treatment of breast tumor was discussed in a recent review paper [57].

## 3 CORRELATION BETWEEN TRANSCRIPTOMIC AND PROTEOMIC DATA

 Until recently, there was an implicit assumption in systems biology literature of the existence of proportional relationship between mRNA and protein expressions measured from a tissue. However, analysis of mRNA and protein expression data from same cells under similar conditions have failed to show a high correlation between the two domains in multiple studies [1-5].

 To analyze the differences between mRNA and protein expressions, we should note that factors having an impact on translational efficiency will have an impact on mRNA-protein correlation. Physical properties of the transcript have a great impact on translational efficiency. One example of such physical property can be Shine-Dalgarno (SD) sequence [58, 59] in prokaryotic transcripts. Transcripts that have weak SD sequence are translated less efficiently. The SD sequence may also be changed by mutation resulting in reduced translational efficiency [60]. Reduction in translation due to mutation in *galE* initiation codon has also been reported [60].

 Another physical property influencing translation is the whole structure of the mRNA. Temperature may change the conformation of mRNA and thus influence translation which was reported in a study for *E. coli* [61].

 In numerous organisms, multiple number of codons can be used to translate same amino-acid which is referred to as ‘codon-bias’ [62]. Codon adaptation index [63] is the measure for codon bias. It is reported that the mRNA-protein correlation is influenced more by codon bias than by SD sequence [64].

 Number of ribosome in a transcriptional unit is called ribosome-density which has a major influence on efficiency of translation. A ribosomal density-mapping procedure to explore ribosome positions along translating mRNAs is described by Eldad *et al.* [65]. mRNAs that entered ribosome for translation (ribosome-associated mRNA) shows better correlation with proteins than typical mRNA expression [66]. Occupancy time of those mRNAs in ribosome also has an impact on translational efficiency which was observed in case of Yeast [67].

 Variability (normalized standard deviation) of mRNA expression level during the cell cycle can also affect the mRNA-protein correlation. This effect is found in cell cycle data by Cho *et al.* [68] which was analyzed by Greenbaum *et al.* [67] and summarized as: "high variability results in high correlation with protein expressions".

 The average half-life of eukaryotic mRNA is reported to be 10-20h whereas the average half-life is 48-72h in eukaryotic proteins in a study in 1989 [69]. From a recent study on mammalian cells, it is reported that, mRNAs are 5 times less stable and 900 times less abundant than proteins and spanned a higher dynamic range [70]. *In vivo* half-life of a protein depends on its amino-terminal residue [71]. Phosphorylation, ubiquitination, and localization of proteins are some post-transcriptional factors which creates variety of half-lives in proteins [72]. Also variation in synthesis and degradation of different proteins creates varied half-lives for proteins which may affect the correlation of protein expression with mRNA. It is also reported that the correlation between half-lives of proteins and mRNAs can be low even when the actual expression level correlation can be higher and also mRNA and protein shares functional properties if they have specific combination of stability (‘stable mRNA stable protein’, ‘stable mRNA unstable protein’, ‘unstable mRNA stable protein’ and ‘unstable mRNA stable protein’) in mammalian cells [70]. The above mentioned stability issues have to be analyzed for modeling dynamic mRNA and protein expression inter-network. 

 Finally, the experimental errors in the type of data extraction approach for protein and mRNA expression brings in extrinsic noise that significantly influences the correlation between mRNA and protein expression.

## 4 OVERVIEW OF DIFFERENT APPROACHES

 In this section, we provide a brief overview of the proposed approaches in literature to jointly analyze transcriptomic and proteomic data. The methods for integrating and modeling transcriptomic and proteomic networks can be categorized primarily into eight different types as discussed next:

1. *Type 1: Union of Transcriptomic and Proteomic Data*: This can be considered as one of the most obvious integration types. Approaches related to this type generally consider a union of two different data sets (proteomic data and transcriptomic data; not from the same sample) and then create a reference data set. The reference data sets have sometimes shown new insights and revealed previously undetected phenomenon or supported a new phenomenon as compared to the individual data-sets. There are a number of approaches related to this [73, 74]. A work on *Bradyrhizobium japonicum* bacteroid metabolism in soybean root nodules by Nathanael *et al.* [49] can be an example of this method. In this study, authors have compiled a reference dataset by combining (union) transcriptomic and proteomic data. Based on the reference dataset, they have discovered significant number of enzymes related to several types of bacterial metabolism that were not present in the dataset from proteomic study alone. Section 5.1 briefly reviews the approach considered by Nathanael *et al*.
2. *Type 2: Extraction of Common Functional Context of Transcriptomic and Proteomic Features*: For various reasons [72], transcriptomic and proteomic data may not have direct overlap in features (here *feature* refers to different genes for transcripts and proteins). But features on transcriptomic and proteomic level might share the same functional context. These functional contexts may refer to different biological processes or pathways in which features from both transcripts and proteins are enriched. In this approach, the common functional contexts are extracted through the analysis of both transcriptomic and proteomic datasets on the level of protein interaction networks. This approach was published in 2010 by Paul *et al.* [75] which is discussed in section 5.2. Authors of this publication also generated *omicsNET* for finding dependency between features of proteomics and transcriptomics.
  A similar kind of approach (functional analysis) was applied for integrating transcriptomic and proteomic evaluation of gentamicin nephrotoxicity in rats by Com *et al.* in 2011 [39]. But the functional analysis was done by GO-Browser ^2^ (an in-house Gene Ontology based annotation tool) with help of Ingenuity Pathway Analysis software^3^. Based on the functional analysis, some gene ontology biological processes were selected which were enriched by the features of the transcriptomic and proteomic dataset with Fisher *p*≤0.05. This integration by functional analysis reveals a putative model of toxicity [39] in the kidney of rats.
3. *Type 3: Topological Networks Approach*: Topological network methods (over-connection analysis, hidden node analysis, rank aggregation and network analysis) have been used to elucidate the common regulators (transcriptional factors and receptors) from two different types of data sets (transcriptomic and proteomic) by Eleonora Piruzian *et al.* [37]. This category of approach refers to locating upstream regulators of mRNA and proteins individually and collecting the common regulators in both the networks for a combined signaling pathway. Topological and network analysis was used in finding individual transcription factors (TF) of mRNAs and Proteins. The TFs that were not common in transcriptomic and proteomic profiles were ignored and the common TFs were used to find the most influential receptors that could trigger maximal possible transcriptional response. Among the receptors discovered from joint analysis, some of them were never reported as *psoriasis* markers in earlier studies while some of them have been reported before. In another recently published study [76], an integrated quantitative proteomic, transcriptomic, and network analysis approach was discussed which also reveals molecular features of tumorigenesis and clinical relapse. Section 5.3 discusses the approach of Eleonora *et al.*.
4. *Type 4: Merging datasets in individual domains*: Type 4 integration merges multiple proteomic data sets into a merged-proteomic data set along with joining multiple transcriptomic data sets into a reference transcriptomic data set. The transcriptomic and proteomic datasets that are merged can be created by different transcriptomic and proteomic profiling respectively. After merging the datasets, correlation analysis is conducted between these 2 merged data-sets and it is shown that the coefficient of correlation is better than the one without merging. Furthermore, specific subsets of the merged data sets can have higher coefficient of correlation. Dov Greenbaum *et al.* [67] used such an approach in their publication in 2003 which is discussed in section 5.4.
5. *Type 5: Missing Value Estimation by non-linear optimization*: This category of integration uses non-linear or linear optimization to predict missing values of proteomic data. It maximizes an objective function to find out the connections between transcriptomic and proteomic networks. However, they do not result in a dynamic model able to predict the abundance of next time point but rather, they are able to predict the protein expression at the same time point. A good example of non-linear optimization is a method described in Wandaliz Torres-Garcia *et al.* [77] for a study of *Desulfovibrio vulgaris* published in 2009. The method is based on stochastic gradient boosting tree (GBT) proposed by Friedman *et al. *[78]. Stochastic GBT optimization technique was also used in a study of *Shewanella oneidensis* in 2011 [79]. Artificial neural network approach was applied to find the missing values of the proteins using the relations between transcriptomic and proteomic data in a separate study published in 2011 [80]. In section 5.5, we briefly review the approach made by Garcia *et al.* [77] in their *Desulfovibrio vulgaris* study.
6. *Type 6: Multiple regression analysis to predict contribution of sequence features in mRNA-protein correlation*: Protein abundance is not only related to corresponding mRNA abundance but also depends on other biological and chemical factors (termed as *covariates*). For this reason, the idea of multiple regression analysis is used to relate characteristics of different covariates of each individual gene with the mRNA-protein correlation. The multiple regression approach can possibly provide a better explanation of protein variability than traditional single regression technique. Effect of multiple sequence feature (one kind of covariate) on mRNA-protein correlation was discussed by Nie *et al.* in 2006 [81] where they have used multiple regression analysis. Example of another linear regression model can be Poisson’s linear regression model which has been used by Lie Nie *et al.* [47] to elucidate the relationship model of transcriptomic and proteomic networks. In section 5.6, we briefly explain the multiple regression analysis used in [81].
7. *Type 7: Clustering Approaches*: Clustering mRNA and protein abundance datasets individually and locating similarities (and hence correlation) between the individual clusters does not produce promising results (as explained in section 5.7). This failure leads to the assumption that concatenating the proteomic and transcriptomic datasets and then clustering the concatenated dataset may not be a good idea either (details in section 5.7). Based on these observations, a new clustering method called coupled clustering was implemented by Rogers *et al.* [82]. Couple clustering creates certain number of proteomic and transcriptomic clusters and provides the conditional probability of a gene to be in a protein cluster given that it is in an mRNA cluster. These conditional probabilities can reveal the relational complexity of mRNA and protein data. Rogers *et al.* used time series transcriptomic and proteomic data extracted under same experimental conditions. Section 5.7 discusses coupled clustering approach. We would want to emphasize that this type of approach is also not a dynamic modeling approach that can provide temporal predictions.
8. *Type 8:* *Dynamic Modeling*: A number of studies reported in the literature have inferred dynamic models ( such as Boolean network, linear models, differential equation models, Bayesian networks etc.) of GRNs from time series transcriptomic data alone. For example, Liang *et al.* [83] used REVEAL algorithm for inference of Boolean network model from time series mRNA expression data. A basic linear modeling has been proposed by D’haeseleer [84]. GRN Models consisting of differential equations was employed by Guthke *et al.* [85]. Validation of inference procedures of GRN was discussed by Edward R Dougherty [86]. Friedman used Bayesian networks to analyze and model gene expression data [87]. Among the existing network models, Bayesian networks can be applied to combine heterogeneous data and prior biological knowledge. For example, Nairai *et al.* [88] used protein-protein interaction network data for refining the Bayesian Network model of the GRN produced by mRNA data alone. Yu Zhang *et al.* [89] used transcriptional factor binding site data and gene expression data (transcriptomic) to model GRN using Bayesian network approach. Werhli *et al.* [90] integrated multiple sources of prior biological knowledge (TF binding location) with microarray expression data to generate a Bayesian network model.

Section 5.8 discusses approach used by Nariai *et al.* [88]. Similar kind of approach was also used by Segal *et al.* [91] where they identified pathways from microarray data and p-p interaction data.

## 5 SPECIFIC EXAMPLES FOR INTEGRATED ANALYSIS CATEGORIES

 In this section, we provide details and specific examples for the eight categories of approaches for joint analysis of transcriptomic and proteomic data discussed in the previous section.

### 5.1 Type 1 Example: Integration of Omics Data Generated Under Symbiotic Conditions

*Bradyrhizobium japonicum* is a gram negative, rod-shaped, nitrogen-fixing bacterium that communicates with its host plant and develops a symbiotic partnership with its host. The host considered in [49] is the soybean plant *Glycine max* ^4^. The complete genome sequence of *Bradyrhizobium japonicum* was identified by Kaneko *et al.* [92] where 8317 potential protein-coding genes were found. 66153 protein-coding loci have been identified in the genome sequence of *Glycine max* ^5^. 

 Nathanael *et al.* [49] built a database by combining the above mentioned 8317 proteins of *Bradyrhizobium japonicum*, 62199 of the above mentioned 66153 proteins of *Glycine max* and 258 contaminating proteins. They searched the combined database to locate the experimental protein extracts of *Bradyrhizobium japonicum* soybean bacteroids. GeLC-MS/MS experimental data was used for this study. A probability-based protein identification algorithm [93] was employed to identify the proteins from mass spectrometry data by searching the sequence database. The use of combined database was beneficial because of the fact that soybean proteins present in the nodule extracts of *Bradyrhizobium japonicum* and *Glycine max* might have symbiotic relations. 2315 proteins in the experimental dataset were also reported to be present in the combined database. 

 The expression of 2780 *B. japonicum* genes in soybean nodules was reported by Pessi *et al.* [94] in a transcriptomic study in 2007. The experimental condition of this genomic study was same as the proteomic study made by Nathanael *et al. *[49]. In both the transcriptomic and proteomic analysis, stringent filtering criteria for normalization procedure were applied. Several statistical analysis tests (Wilcoxon rank-sum and the student t-test with a P-value threshold of 0.01) based on numerous biological replicates were applied in the microarray based transcriptomic study to prevent erroneous conclusions. The use of transcriptomic expression profiling alone has some limitations (such as limitations of the array i.e. not all the genes are present in the array, concealment of true expression levels due to bias of the probe set, etc.) which are also somehow true for using only proteomic expression profiling. Thus, the authors chose to integrate (through a simple union method) both the data sets and use this list of genes as reference data set for bacteroid expression. In total, 3587 transcriptomic (A) and protein (B) expressions in soybean bacteroids were recorded in the union set (*AUB*). The number of elements in the set *A*∩*B* was 1508. 807 proteins were identified to be expressed by only the proteomics approach (*B*−*A*) and 1272 genes that have been identified as expressed only in the transcriptomic study (*A*−*B*). Among the set *A*−*B*, 47 were RNAs (45 tRNAs, rnpB, ssrA2) and the remaining 1225 were protein encoding genes Fig. (**1**). Summarizes the method of integration.

 A list of 15 gene function categories^6^ were observed for the 3 different datasets: *(i)* the dataset X by Kaneko *et al.* [92] consisting of 8317 protein encoding genes, *(ii)* the reference datasets *AUB* consisting of 3540 (3587−47) protein encoding genes and *(iii)* the dataset *X*−(*AUB*) consisting of 4777 protein encoding genes i.e. the protein encoding genes that are not detected by Nathanael *et al.* [49]. The number of genes present in the 3 datasets for each category was detected. The number of genes/proteins present in each category was divided by the total number of genes/proteins in that data set and thus relative frequency of each category for the 3 datasets was established. In 4 among the 15 categories, it has been observed that the relative frequency in the reference dataset (*AUB*) is less than the relative frequency in the Kaneko dataset (*X*). This means that in only 4 categories, the reference dataset *AUB* was less capable to represent that category than the dataset *X*.

 The reference dataset revealed novel insights regarding some aspects of bacterial metabolism (e.g. Nitrogen metabolism, Carbon metabolism, Nucleic Acid metabolism) and also regarding translation and post-transcriptional regulation. For example *(i)* some key regulator proteins (e.g. GlnA, GlnB, GlnK and GlnII) of N metabolism was identified in the reference dataset. *(ii)* The authors reported that all the enzymes related to C4 metabolism were detected for the 1st time as well as almost the entire set of gluconeogenesis related enzymes was identified in the combined reference data set. *(iii)* In a study of global protein expression pattern of *Bradyrhizobium japonicum* bacteroids by Sarma *et al.* [95], nucleic acid metabolism related proteins were reported to be lacking in the total protein expression pattern of the nodule bacteria. But, in this study, authors have found almost all enzymes related to *de novo* nucleoside and nucleotide biosynthesis either in the gene or in the protein level or in both. *(iv)* The reference dataset also comprises a large number of proteins related to transcriptional and post-transcriptional regulation. Also, the enzymes related to protective response (to reactive oxygen species) under stress were also discovered in the reference dataset. In this context, it can be mentioned that, glutathione is crucial for biotic and abiotic stress management for plants, thus, detecting all the enzymes required for glutathione synthesis and reduction strengthens the richness of the reference dataset. Very few enzymes in metabolic pathways were discovered in the dataset derived from the proteomic study alone (dataset *B*). Thus the combination of data can provide novel insights for carbon and nitrogen metabolism.

### 5.2 Type 2 Example: Functional Analysis of Transcriptomic and Proteomic Data

 An approach for linking transcriptomic and proteomic data on the level of protein interaction network has been discussed in a recently published paper [75]. Transcriptomic and proteomic datasets characterizing the chronic kidney disease (CKD) have been used to illustrate the procedures for integrating omics profile at the level of protein interaction networks. 

 Three publicly available studies on CKD by Schmid *et al.* [96], Baelde *et al.* [97] and Rudnicki *et al.* [98] are used for identifying deregulated features on the mRNA level. 697 differentially regulated genes were selected from the 3 studies creating the transcriptomic dataset. The proteomic dataset was extracted from the online database HUPDB *v*2.0 ^7^. A total of 192 samples were used and after comparing with the CKD and healthy references, 37 proteins were identified as differentially abundant. HUPDB was selected as the only source to avoid heterogeneity of datasets. Swiss-prot annotation tool was used in this study (as HUPDB uses Swiss-prot names as identifiers) to map the proteins to the gene symbols. Swiss-Prot [99] or UniprotKB is a protein sequence database which provides all known relevant information about a particular protein. The details about Swiss-prot entry annotation can be found in *http://www.uniprot.org/faq/45*. 

 The following five different analysis procedures have been discussed and compared extensively in this study to elucidate the correspondence between transcriptomic and proteomic data: *(i)* Direct feature overlap, *(ii)* Functional overlap; here features (genes) related to different biological processes that are found in transcriptomic or proteomic datasets or both has been analyzed, *(iii)* Joint pathway analysis, *(iv)* Protein dependency graph analysis and *(v)* Direct edges between transcripts and proteins.

#### (i) Direct feature overlap:

Here ‘feature’ refers to different genes for transcripts and proteins. Features present in both the transcriptomic and proteomic lists were identified. Genes of 4 proteins^8^ (out of 37) were also reported to be differentially expressed in the transcriptomic dataset. This overlap was confusing as only 1 of them was upregulated in both the datasets but the other 3 shows upregulation in one dataset and downregulation in another. 

#### (ii) Functional Overlap:

PANTHER (Protein ANalysis THrough Evolutionary Relationships) classification system [100, 101] classifies proteins and their genes to facilitate high-throughput analysis. The classification of proteins were done according to ‘family and subfamily’, ‘molecular function’, ‘biological process’ and ‘pathway’. Multiple gene lists can be uploaded in the PANTHER system and jointly compared against a reference dataset to look for under and over represented functional categories based on either ‘chi-square test’ or ‘binomial statistics’ tool. Here, the authors used PANTHER to identify enriched biological process. They have used fully annotated set of human genes as a reference dataset and used chi-square test with a p-value less than 0.05 to identify significantly enriched or depleted biological processes. They had identified 27 biological processes ^9^ that are relevant to the transcriptomic and proteomic datasets. Four of the processes were found to be enriched by both the transcriptomic and proteomic feature set. Other processes were enriched by either transcriptomic or proteomic features.

#### (iii) Joint Pathway Analysis:

The laboratory of Immunopathogenesis and Bioinformatics (LIB) developed the DAVID (the Database for Annotation, Visualization and Integrated Discovery) tool [102] to provide functional interpretation of large lists of genes derived from different genomic studies. KEGG pathway database is used as a repository for applying DAVID tool in this study. Seven pathways ^10^ are found to be significantly enriched in deregulated transcripts and/or proteins using Fisher exact test with p-value less than 0.05. Among these 7, 3 pathways are enriched with both the transcriptomic and proteomic features and 4 pathways are enriched with either transcriptomic or proteomic features. 

#### (iv) Protein Dependency Graph Analysis:

PANTHER and KEGG do not cover all the features found in transcriptomic and proteomic dataset used in this study. So, the authors had developed undirected protein interaction network omicsNET [103] which includes all protein encoding genes as nodes in the network. It has edges between the nodes with edge weights referring to dependency measures between the pair of nodes. The dependency measures were determined using Gene Expression Omnibus Human Body Map, the MicroCosm database, Gene Ontology data on molecular processes and functions, PANTHER, KEGG and IntAct databases. The research team found 65 strong dependencies in omicsNET between the features of transcriptomic and proteomic dataset of this study. The features that are involved in the dependency graph include 21 features from transcriptomic dataset, 21 features from proteomic dataset and 2 features from both. Also, dependencies between the features related to blood coagulation cascade was analyzed using omicsNET on different edge weight values. 

#### (v) Direct edges between transcripts and proteins:

MAPPER^11^ (Multi-genome Analysis of Positions and Patterns of Elements of Regulation) is a platform for identifying transcription factors binding sites (TFBSs) in multiple genomes [104]. Binding sites of 4 transcription factors were identified in ORF (open reading frame) regions of the 37 proteins using MAPPER. 2 of these 4 transcription factors shows upregulation and 2 shows downregulation in mRNA level. At least 1 of these 4 transcription factor binding sites are present in 13 proteins of the protein dataset. This fact reveals some direct edge between transcripts and proteins.

 Use of direct feature overlap gave only limited and ambivalent results. But this limited overlap increases when enriched biological processes were identified based on transcriptomic and proteomic datasets. Several biological processes were identified significantly enriched with both transcriptomic and proteomic features. Mapping transcriptomic and proteomic features on different KEGG pathways also reveals significant involvement of both transcriptomic and proteomic features; three KEGG pathways are found to be enriched with them. And finally using omicsNET for overcoming the shortcomings of PANTHER and KEGG gives significant outcome for understanding the interactions of the transcriptomic and proteomic network. 

 The main advantage of functional analysis of transcriptomic and proteomic data is that different pathways and processes for the genes under analysis become evident. Although dependency measures can be found between transcripts and proteins from omicsNET, the main shortcoming is that it cannot create a dynamic model involving transcripts and proteins.

### 5.3 Type 3 Example: Comprehensive Meta-Analysis

Topological network analysis was used to reveal the similarities and differences between transcriptomic and proteomic-level perturbations in *psoriatic lesions* in a recent study [37]. The transcriptomic data related to psoriatic lesions contains 462 over-expressed transcripts and the proteomic data contains 10 abundantly expressed proteins. Unlike most of the studies, this study shows good consistency between the proteomic and transcriptomic dataset as 7 out of the 10 protein encoding genes were also over-expressed in the transcriptomic dataset. But the significant difference in the magnitudes of the 2 dataset hinders direct correlation analysis. Rather than analyzing correlation between them, the authors used topological network approach to discover regulatory transcription factors, receptors and their ligands to reconstruct the network between them. Their approach produced biologically meaningful results and revealed unknown regulatory receptors that may be related to psoriatic lesions.

The methods used in the study include *(i)* Over-connection analysis *(ii)* Topological analysis: hidden node analysis *(iii)* Rank aggregation and *(iv)* Network analysis.

#### Interactome Overconnectivity Analysis:

It is assumed in this analysis method that the expression values of transcripts and proteins follow hyper geometric distribution. The method for finding overconnected regulators (transcription factors) of a target dataset is described in the supplementary material of publication by Nikolsky *et al.* [105]. This overconnection analysis mainly ranks transcription factors (assign a score to it). The score or significance of a transcription factor (taken from global gene database or manually curated gene database like MetaCore ^12^) is a function of ‘hypergeometeric distribution probability mass function’ [37, 105].

#### Hidden Node Analysis:

The complete algorithm for hidden node analysis ^13^ has been discussed by Dezso *et al.* [106]. Here we’ll try to demonstrate what it actually does by a very simple example. Fig. (**2**) demonstrates 4 genes (nodes) x_1_, x_2_, x_3_ and x_4_ which are over-expressed or abundant in a transcriptomic or proteomic dataset. Hidden node analysis reveals node x_5_ which was not present in the experimental data but is the key to regulate downstream effects of targets x_2_, x_3_ and x_4_. The members of the hidden nodes may come from a global database or manually curated database like MetaCore.

#### Rank Aggregation:

When we have multiple ordered lists, rank aggregation approaches can be used to combine them into a single list. Rank aggregation can be formulated as a optimization problem [107] with the objective function being the weighted sum of distances of the original list from the combined list $O\left(L\right)=\sum_{i=1}^{m}w_{i}d\left(L,L_{i}\right)$ where *L* is the combined list and *L_i_* denote the individual lists and *d* is a distance function. An example of the distance function is the Spearman footrule distance which is the absolute sum of the differences in the ranks of the unique elements of the individual list and the combined list. The optimization can be carried out using approaches such as Cross-Entropy Monte Carlo stochastic search [108] or genetic algorithms.

#### Network Analysis:

A typical *network analysis* helps to select biologically connected meaningful sub-networks with relevant objects. For example, 10 over-expressed proteins were taken as relevant objects in this study and published literature was used to come up with regulatory transcription factors, receptors and kinases.

For the integrated study, 10 common transcription factors (top ranked) of transcriptomic and proteomic data types were identified using overconnection analysis and hidden node analysis. But before this step, 20 TFs were identified for each data type using topology analysis and 5 TFs were identified using network analysis approach. These 10 common nodes in each data type shows resemblance with the TFs found from literature search. Next, hidden node algorithm was used to find the most influential 44 membrane receptors that are present in the same signaling pathway with one of the 10 common TFs and whose genes or corresponding ligands were 2.5 fold (or greater) over expressed in the experimental data. For the hidden node analysis to find the 44 receptors, the target set consisted of 462 differentially expressed genes. Among the 44 receptors, 22 were previously reported to be related to psoriatic lesions. 14 of them shows possible but not confirmed relation to psoriatic lesions and rest of the 8 receptors were not reported before. 

Thus, topological analysis can be applied to identify common regulators from two different datasets. The common regulatory machinery can be applied to arrive at a biologically meaningful signaling pathway that can be verified through new experiments or existing reported results in literature.

### 5.4 Type 4 Example: Merging Datasets into Meta-Datasets

In a review paper published in 2003, Dov Greenbaum *et al.* [67] discussed the results of the comparisons of different approaches on correlating mRNA expression with protein abundance. The authors focused on yeast.

At first, they created a ‘reference mRNA dataset’ using *iterative combination of different datasets* (as shown in Algorithm 1) that had been earlier discussed by Greenbaum *et al.* [38] in 2002. They used 4 different datasets from 4 different studies: 3 of the datasets [109-111] used Affymetrix chips and the remaining dataset [112] used the SAGE method. The 4 datasets were merged using algorithm 1 to create the ’mRNA reference dataset’. 

As a following step, four proteomic datasets were from Gygi *et al.* (2DE-1 dataset [3]), Futcher *et al.* (2DE-2 dataset [113]), Washburn *et al.* (MudPit-1 dataset [114]) and Peng *et al.* (MudPit-2 dataset [115]) were merged to create a *reference protein dataset*. The merging technique is illustrated in algorithm 2. 

The correlation coefficient (r) of the *reference mRNA dataset* and the *reference protein dataset* was derived to be *r*=0.66. Correlation for some smaller functional categories of proteins was also calculated and a mix of higher correlation values ^14^ as well as lower correlation values ^15^ was reported.

The ideas of merging mRNA datasets (Algorithm 1) and merging proteins (Algorithm 2) have some sort of similarity. The mRNA merging technique uses one of the mRNA datasets it is merging as a reference that is used to find the regression parameters while the protein merging technique uses the *reference mRNA dataset* in this regard. The values of α and β is an important factor in mRNA merging technique while the quality ranking of the protein datasets have important influence on merging the protein datasets. The order 2*DE*−1>2*DE*−2>*MudPit*−2>*MudPit*−1 is used as quality ranking that depends on the confidence level of the accuracy of the datasets.

#### Algorithm 1:

Algorithm for generating the mRNA reference dataset

- Let *X*_1_, *X*_2_....*X*_n_ define different mRNA expression datasets from *n* different experiments using Gene Chips. Let the last dataset *X*_n_ denote the dataset with highest accuracy. Let *X*_n_ be denoted by *X_H_*. The dimension of these datasets may not be equal i.e. each dataset does not contain mRNA expression of all N genes that are present jointly in the *n* datasets. Here *X_j_(i)* will denote the mRNA expression of the *ith* gene in the *jth* database *i*∈1,2,...*N* and *j*∈1,2,...*n*.
- Initialize *MergedData*=[]
- Set α=0.15. This can be changed.
- ***for** j*=1:*n*−1
  - Find the common genes present in *X_j_* and *X_H_*
  - Find the parameter *p*_1_ and *p*_2_ while minimizing the expression $\sum_{k}{\left(p_{1}X_{j}^{p_{2}}\left(k\right)-X_{H}\left(k\right)\right)}^{2}$ where *k* is the member of common gene set in *X_j_* and *X_H_*
  - **for** i=1:N
***if ***gene *i* is present in both *X_j_* and *X_H_* and $\frac{\left|p_{1}X_{j}^{p_{2}}\left(i\right)-X_{H}\left(i\right)\right|}{p_{1}X_{j}^{p_{2}}\left(i\right)-X_{H}\left(i\right)}<\alpha \mathrm{then Merged Data}\left(i\right)=\frac{p_{1}X_{j}^{p_{2}}\left(i\right)-X_{H}\left(i\right)}{2}$
    *** elseif ***gene *i* is present only in *X_j_** then***
*MergedData(i)=X_j_(i)*
*** elseif*** gene *i* is present only in *X_H_* ***then***
*MergedData(i)=X_H_(i)*
*** elseif*** gene *i* is present neither in *X_j_* nor in *X_H_* ***then***
    Do nothing. mRNA expression for *ith* gene will not be present in the *MergedData*
***endif***
***endfor***
  - Make the *MergedData* as the *X_H_* for next iteration
***endfor***
- Let S denote the SAGE data.
- Set β=16.
- **for** i=1:N
***if*** gene *i* is present in both *MergedData* and *S* and *MergedData*(*i*)>β and *MergedData*(*i*)<*S*(*i*) ***then*** *MergedData*(*i*)=*S*(*i*)
***endif***
***endfor***

#### Algorithm 2:

Algorithm for creating Protein reference dataset

- Let *P*_1_, *P*_2_.....*P_2_* Denote protein expression datasets from *n* different experiments. Let the last dataset *P_n_* denote the dataset with the highest confidence level in its accuracy. The dimension of these datasets may not be equal i.e. each dataset does not contain protein expression of all N genes that are present jointly in the *n* datasets. Here *P_j_(i)* will denote the protein expression of the *ith* gene in the *jth* database. *i*∈1,2,...*N* and *j*∈1,2,...*n*
- Let *M* denote the mRNA reference dataset created using algorithm 1
- Find the parameters *a_j_* and *b_j_* while minimizing the expression $\sum_{k}{\left(a_{j}M^{b}j\left(k\right)-P_{j}\left(k\right)\right)}^{2}$ where *k* is the member of the common gene set in *P_j_* and *M* and *j*∈1,2,....*n*.
- Find *Y_j_=a_j_M^b^j* for all *j*ϵ1,2,...*n*. This is the transformation of the protein databases into mRNA reference dataset.
- Find ${\overset{\wedge }{P}}_{j}=a_{n}{\left(\frac{Y_{j}}{a_{j}}\right)}^{\frac{b_{n}}{b_{j}}}$ for all *j*∈1, 2,...*n*. This is the inverse transform into the protein space using parameters of the most accurate set *P_n>_*.
- Combine the set ${\hat{P}}_{1},{\hat{P}}_{2},...{\hat{P}}_{n}$ into *MergedData*. To do this, order of confidence for the original datasets will be used i.e. if expression for gene *i* is present in multiple datasets, then the value of that gene in *MergedData* will come from the dataset with the highest confidence.

The authors’ argument in support to their proposed protein merging technique is that the resulting protein reference dataset is a better quantitative and a representative one that is easier to compare with the mRNA expression dataset and is supported by the increased correlation coefficient in some functional categories. Thoughtful scaling techniques were applied to avoid biases in the datasets and in case of multiple possibilities of entering values into the reference dataset from individual datasets, a plausible method of quality ranking was used. So, the ultimate success can be viewed as finding higher correlations among different functional categories. The lower correlation in a functional category reflects heterogeneity in that category. 

 We should note that often due to different half-lives of mRNAs and Proteins, we will observe lack of correlation between transcriptomic and proteomic datasets measured at the same time under symbiotic conditions. Merging of multiple datasets normalizes and integrates the expression values which are more likely to have a higher correlation. But the emphasis on correlation can be sometimes misleading as correlation measures the linear dependence and a perfect non-linear dependence between two variables can be ignored by correlation analysis. 

### 5.5 Type 5 Example: Non-Linear Optimization Model to Integrate Transcriptomic and Proteomic Data

Garica *et al.* [77] implemented stochastic Gradient Boosting Tree (GBT) approach to infer non-linear relationships between mRNA and protein expression data and estimate the missing protein expressions using the generated relationship. They had mRNA expression data of around 3500 genes and protein expression data of around 800 genes of *Desulfovibrio vulgaris*. After locating the non-linear relationship and the missing protein expression values, they validated the result using knowledge from literature. The total procedure is shown in Fig. (**3**).

#### Gradient Boosting Tree Method:

Gradient boosting approach was proposed by Jerome H. Friedman in 2001 [116]. It is a nonlinear regression technique that produces a prediction model in the form of decision trees. Some modification of this method has been proposed by Friedman in 2002 [78]. The method uses a training set (x_1_,y_1_), ..., (x*_n_*,y*_n_*) that tries to find an approximation Ĝ(*x*) to a function *G*(*x*) that minimizes the expected value of some specified loss function φ(*y*,*G*(*x*)).

$$\overset{\wedge }{G}\left(x\right)=\underset{G\left(x\right)}{\mathrm{argmin}}E_{y.x}\varphi \left(y,G\left(x\right)\right)$$

Several loss criteria can be used including least squares: $\varphi \left(y,G\right)={\left(y-G\right)}^{2}$ least absolute deviation: $\varphi \left(y,G\right)=\left|y-G\right|$ etc. Garcia *et al.* used least squares criteria.

The total procedure was divided into α iterations. Each iteration is called a regression tree. In each tree, pseudo residuals of the dependent variable (here, proteomic expression) of the training dataset were located. Then the total input space has been divided into β disjoint regions using least square splitting criterion [87]. These regions are called the leaves of the tree. In each region, a multiplier value η_αβ_ is calculated here α denotes the α*^th^* tree and β denotes the β*^th^* leaf of that tree.

Thus, the approximation function Ĝ(*x*) mainly depends on the splitting variables and splitting points for each tree and also on the value of η in each leaf. Using the training dataset, these variables are estimated and used in future prediction.

Algorithm 3 demonstrates how Gradient Boosting Tree method works. Here the modified version by Friedman which he called *TreeBoost* is shown. Friedman introduced another parameter ξ (0<ξ<1) which controls the learning rate of the algorithm. This is called ‘shrinkage parameter’. In each tree of each iteration, the value of η is multiplied by the value of ξ. According to Friedman [116], choice of the value of ξ is important for the performance of the algorithm; small values cause less prediction error. 

#### Stochastic Gradient Boosting Tree:

A small change in the gradient boosting tree method can make it stochastic. For Stochastic GBT, a random subset of training dataset is used in each iteration rather than using the total training dataset. According to Friedman, the incorporation of randomness and the use of training data subset improve the performance of prediction as well as reduce computational complexity.

#### Algorithm 3

Algorithm for implementation of Gradient Boosting Tree method (modified version ‘TreeBoost’)

- $$G_{0}\left(x\right)=\underset{g}{\mathrm{argmin}}\sum_{i=1}^{n}\varphi \left(y_{i},g\right)$$
  ***for ***a=1:α
  - Compute pseudo residuals of the dependent variable of the training dataset:
    $$\tilde{y_{\mathrm{ia}}}=-{\left[\frac{\partial \phi \left(y_{i},G\left(x_{i}\right)\right)}{\partial G\left(x_{i}\right)}\right]}_{G\left(x\right)=G_{a-1}\left(x\right)}$$
    for *i*=1,...,*n*
  - Divide the training data space into β different regions *R*_1a_, *R*_2a_...*R*_βa_ using pseudo residuals. Least square splitting criterion is used to split the region.
  - Compute multiplier η_ba_ for each region *b* (*b*∈0,1,2...β) by solving the following optimization:
    $$\eta_{\mathrm{ba}}=\underset{\eta }{\mathrm{argmin}}\sum_{x_{i}\in R_{\mathrm{ba}}}\varphi \left(y_{i},G_{m-1}\left(x_{i}\right)+\eta \right)$$
  - Update the model:
    $$G_{m}\left(x\right)=G_{m-1}\left(x\right)+\xi \sum_{b=1}^{\beta }\eta_{\mathrm{ba}}I\left(x\in R_{\mathrm{ba}}\right)$$,
    where I(.) is the indicator function.
    **endfor**
- Output *F*_α_(x).

#### Validation process:

 The validation of the predicted missing values is important for performance analysis. Garcia *et al.* used existing biological knowledge to validate their results. The biological knowledge of *Desulfovibrio vulgaris* includes *(i)* functional categories of all genes (20 categories found from Comprehensive Microbial Resource [117]), *(ii)* sequence length, protein length, molecular weight, guanine-cytosine and triple codon counts of all gene, *(iii)* a total of 609 operons of *Desulfovibrio vulgaris* consisting of 2 to 13 genes in each operon *(iv)* Regulons of *Desulfovibrio vulgaris* and *(v)* 92 metabolic pathways (KEGG pathways) for microbial genomes. 

 The Coefficient of Variation (CV) for different operons and regulons are calculated using the predicted values of proteins by dividing the standard deviation (SD) by mean expression value of each operon/regulon/pathway group. Let an operon or a regulon or a pathway group has *n* genes in it; a random set consisting *n* genes was created and CV (*CV_random_*) was calculated for that set of genes. This process was repeated for 1000 times for each operon/regulon/pathway and mean for *CV_random_* was calculated. The mean *CV_random_* was then compared against the CV of the original operon. The idea is that the variability of genes in operons or regulons will be less than the variation of random set of genes because the genes in an operon or regulon are supposed to be expressed together and are relationship in their expressions. If the CV of an operon is found to be less than the mean *CV_random_*, then it is concluded that, the predicted values of that operon is somehow close to accurate. The results found by Garcia *et al.* shows that a large portion of the operons/regulons/pathway groups indeed has less variability than the variability of randomly created groups of gene. Another measure used in this study for understanding the less dispersion of gene expressions in operons/regulons/pathways is ‘percentile score’. It’s the percentage of 1000 set of random genes for each operon/regulon/pathway group which have CV values (*CV_random_*) less than the CV of original operon/regulon/pathway. Thus, the percentile score is expected to be less to prove that the variability in expression of genes in an operon/regulon/pathway is less dispersed than the random set of genes. 

 Correlation between protein and mRNA expression was measured for each operon/regulon/pathway groups and also for all the genes. It was found that the correlation was stronger in most of the individual operons and pathway groups than the correlation for all genes. Small fraction of the regulon groups showed better correlation.

 The method applied for the validation process of this study clearly gives an idea about the overall prediction accuracy but does not guarantee a good prediction. A poor prediction for all the genes in an operon might produce *CV* less than the mean *CV_random_* if the overall predictions for other operons are also similarly poor. A different option for validation may be combination of this approach and incorporation of a testing dataset for cross-validation. The testing dataset will be a set of mRNA and protein datasets other than the training data whose actual values are also known. However, it will obviously reduce the size of training dataset. The predicted values can be compared with the original values along with the method of validation using biological knowledge.

### 5.6 Type 6 Example: Linear Regression Model to Integrate Transcriptomic and Proteomic Data

In a study on *Desulfovibrio vulgaris* by Nie *et al.* [81], the effect of sequence features in different translational stages on the correlation of mRNA expression and protein abundance has been discussed. Multiple regression analysis that has been previously discussed in another paper by Nie *et al.* [118] was applied to predict the contribution of different sequence features on the correlation of mRNA and protein abundance. 

#### Sequence features in translational stages:

A sequence feature ^16^ can be defined as an entity or data located in DNA or RNA sequences that are responsible for different biological phenomena. For example, Shine Dalnargo sequence is a sequence feature which is mainly a ribosomal binding site in mRNA and it helps the ribosome to start synthesis of protein. Other examples of sequence features can be start codon, stop codon, codon usage etc. In prokaryotes, translation can be divided into 3 stages: initiation of translation, elongation of translation and termination of translation. Lithwick *et al.* [64] demonstrates hierarchy of sequence features related to prokaryotic translation. Shine Dalgarno sequences, start codon identity and start codon context are examples of *initiation* feature; codon usage and amino acid usage are examples of *elongation* feature; stop codon identity and stop codon context are examples of *termination* feature. 

#### Multiple Regression Analysis:

 A simple regression analysis can be expressed through the following equation:

*Protein_i_=A+B×mRNA_i_*

 The target is to find *A* and *B* that relates the two variables. Here, *mRNA_i_* and *Protein_i_* are logarithm of the mRNA and protein value of gene *i* respectively. Nie *et al.* [118] reported that only 20−28% (Pearson correlation coefficient *R*^2^) of protein variability can be captured by simple regression analysis. This is because, protein abundance is not only related to corresponding mRNA abundance but also depends on other different biological and chemical factors (termed as ‘covariate’). So multiple regression analysis is required which can be expressed as:

$${\mathrm{Protein}}_{i}=A+\left({\mathrm{mRNA}}_{i}\times B\right)+\sum_{j=1}^{k}\left(B_{j}\times {\mathrm{Covariate}}_{\mathrm{ij}}\right)$$

where *Protein_i_* and *mRNA_i_* are the protein abundance data and the mRNA expression level for the *i*^th^ gene respectively. *Covariate_ij_* refers to the *j^th^* covariate of the *i^th^* gene. *B_j_* represents the slope for the *j^th^* covariate. Nie *et al.* [118] found that 52−61% (Pearson correlation coefficient, *R*^2^) variability of protein can be captured by this multiple regression analysis.

In this study of effect of sequence features, Nie *et al.* [81] used the sequence features in different translational stages as covariates and performed multiple regression analysis to locate the sequence features that has the highest effect on the mRNA-protein correlation. They have done multiple regression analysis for each type of sequence feature (i.e. sequence features related to initiation stage, elongation stage and termination stage) separately and also for a combination of sequence features. The results showed that the sequence features are significantly responsible for the variation in mRNA-protein correlation. And also mRNA-protein correlation was affected the most by elongation stage features. The method of finding the effect of covariates in mRNA-protein correlation using one of the 3 datasets can be visualized by the flowchart shown in Fig. (**4**).

Three different datasets containing transcriptomic and proteomic measurements were used in this sequence feature analysis of *Desulfovibrio vulgaris*. The three datasets were expression levels under three different growth conditions (lactate, formate and lactate-stationary). Partial correlation coefficient $R_{p}^{2}$ was used to find the contribution of specific sequence features in the variability. The partial correlation coefficient can be interpreted as:

$$R_{p}^{2}=\frac{R_{2}^{2}-R_{1}^{2}}{1-R_{1}^{2}}$$

where $R_{1}^{2}$ is the Pearson correlation coefficient using only simple regression; $R_{2}^{2}$ is the Pearson correlation coefficient when multiple regression model is used with sequence features included. Standard F-test [119] was used to examine the significance (P-value for the F-test) of each covariate.

 The $R_{p}^{2}$ for different sequence features varied; for ‘SD sequence’: 1.9%−3.8%, for sum of ‘start codon’: 0.1%−0.7%, for ‘start codon context’: 0.3%−2.6%, for sum of ‘codon usage’: 5.3%−15.7%, for sum of ‘Amino acid usage’: 5.8%−11.9%, for sum of ‘stop codon’: 1.3%−2.3%, for sum of ‘stop codon context’: 3.7%-5.1%.

 The sum of the individual $R_{p}^{2}$ values for all the sequence features are ranged in 21.8%−39.8% where the $R_{p}^{2}$ for sequence features together in a single multiple regression ranged in 15.2%-26.2%. 

 So, the analysis proved that, among multiple sequence features, ‘amino acid usage’ and ‘codon usage’ are the top factors that affect the mRNA-protein correlation. The results were validated by conducting similar analysis where sequence features were kept same for all the genes but protein values were randomly assigned to the mRNA values. The resulting P-value in this validation stage analysis was found to be less than the original P-values which indicates better statistical significance of the model found.

 Pointing out the factors affecting the mRNA-protein correlation was the major contribution of this study. These results can be utilized in creating robust model for mRNA and protein expression values. This is another proof of the fact that only mRNA expression does not necessarily have the power of predicting the protein expression. Complex biological factors such as sequence features related to translational stages should have a significant role in their prediction procedure.

### 5.7 Type 7 Example: Correspondence Between Transcriptomic and Proteomic Expression Profiles Using Coupled Cluster Models

 The mRNA or protein expression of a random set of genes is likely to show multiple different levels of expression, but genes involved in similar functions or having similar effects on cellular regulation might show close expression levels. A mixture model [120] generally clusters such datasets into a predefined number of sub-sets in an unsupervised manner. For example, Gaussian mixture model is an unsupervised clustering algorithm where it is able to create soft boundaries among the clusters, i.e. points in the space can be present in any cluster defined by a given probability. This is primarily a mixture of a certain number of Gaussian distributions with unknown parameters where each Gaussian distribution fits its corresponding cluster. Estimation maximization (EM) algorithm [121] is used to find the parameters of the Gaussian distribution and the cluster probabilities.

 Simon Rogers *et al.* [82] proposed a coupled mixture model to investigate the correspondence between transcriptomic and proteomic expressions. The dataset consisted of transcriptomic and proteomic profiles of 542 human genes from the Human Mammary Epithelial Cell line (HMEC). Measurements were taken between 0 and 24*h* after the cells were stimulated with Epidermal Growth Factor (EGF). There were a total of 6 transcriptomic (mRNA) measurements (1 hr, 4 hr, 8 hr, 13 hr, 18 hr, 24 hr) and 7 proteomic (proteins) measurements (15', 1 hr, 4 hr, 8 hr, 13 hr, 18 hr, 24 hr).

 The mRNA and protein datasets were clustered individually using Gaussian mixture model and the similarity between the two sets of clusters were determined by standard Rand index [122]. The standard Rand index is ranged from 0 to 1 where 1 denotes that the two cluster sets are exactly same. It was observed in the study that the two cluster sets showed very little similarity. The large dissimilarity suggested that if the two datasets were clustered after concatenating them into a single dataset, the number of clusters could have been as large as 20*15=300 which was impractical as the total number of genes was 542. Also, by comparing gene ontology (GO) enrichment analysis, it was discovered that the individual clustering produced different biologically meaningful clusters which were lost when clusters were created after concatenation. The failure of individual and concatenated clustering lead to the implementation of coupled mixture models described in this study. The ideas of clustering individually and clustering after concatenation are illustrated in Figs. (**5** and (**6**) respectively.

 In coupled mixture modeling, the mRNA dataset was clustered into ‘*U*’ different clusters with *p*(*u*)(*u*∈1,2...*U*) denoting the probability that mRNA expression of a gene belongs to the *u^th^* cluster. Similarly protein dataset was clustered into ‘*V*’ different clusters with *p*(*v*)(*v*∈1,2...*V*) denoting the probability that protein expression of a gene belongs to the *v^th^* cluster. The joint probability can be described as *p*(*u*,*v*)=*p*(*u*)*p*(*v*|*u*) where *p*(*v*|*u*) is the parameter that provides the relationship between mRNA expression and protein level.

 The EM algorithm was used to maximize a log-likelihood function (equation 1 in supplementary material of [82]) to infer the desired parameters. The number of clusters (U and V) in each dataset was derived to be *U*=15 and *V*=20 using Bayesian Information Criterion (BIC, proposed by Gideon E. Schwarz [123]). Fig. (**7**) demonstrates the coupled mixture model.

 The values of *p*(*v*|*u*) can unravel important information about the complexity of the relationship between mRNA and protein expressions. For example, the protein cluster *v*=4 had a total of 19 proteins in it, 18 of those were ribosomal proteins. There were 7 mRNA clusters which had positive *p*(*u*|*v*=4) within the protein cluster *v*=4. The most connected mRNA cluster with this protein cluster was the cluster *u*=3 because *p*(*u*=3|*v*=4)=0.3653 (which was the highest among all *p*(*u*|*v*=4)). If we look at the other protein clusters which are related to this mRNA cluster *u*=3, we’ll see that, there are 14 protein clusters which have positive *p*(*v*|*u*=3). This complex set of information suggests that indeed the relationship between mRNA and protein expression is a complex one; this decision could not be made with the results of individual clustering and concatenated clustering technique. The inference of complex relationships from those conditional probabilities remains open; may be use of more biological knowledge about the involvement of different sets of mRNA and proteins in different biological processes will reveal the relationship more clearly. Rogers *et al.* concentrated on three biological phenomena and others are still open problems. The three biological phenomena they dealt with are: *(i)* conserved behavior of ribosomes occurring at the protein level, *(ii)* discovering interesting set of genes involved in cell-adhesion and *(iii)* The role of TCP-1 as a protein folding machine.

 The choice of the model in each component of the mixture is not limited to Gaussian. Other models can be used to construct mixture models such as ordinary differential equation model used by Chudova *et al.* [124] and B-splines used by Luan and Li [125].

 We should note that the mRNA and protein expression of 542 genes used in Rogers *et al.* was a subset of the original dataset that had a lot of missing values for proteomic expression. The genes that had both transcriptomic and proteomic values present were used in the study. An interesting idea may be to use missing value prediction method described in section 5.5 (method by Garcia *et al.* [77]) to complete the original dataset and use that in this study. Combining the approaches by Garcia *et al.* [77] and Rogers *et al.* [82] can possibly improve mRNA and protein correspondence when missing values possess a significant issue.

### 5.8 Type 8 Example: Dynamic Models

 Nariai *et al.* [88] used cell cycle microarray data [126] of *Saccharomyces cerevisiae* and 9030 protein-protein interaction data derived from MIPS database [127] to construct a Bayesian network model. The authors proved that the use of p-p interaction data had refined the estimated gene network produced by using only microarray data. The algorithm used to construct the network can be simply illustrated by the flowchart showed in Fig. (**8**). The algorithm is designated as the greedy hill-climbing algorithm.

 In the greedy hill-climbing algorithm, each network was evaluated by Bayesian network and Nonparametric Regression Criterion (BNRC) score [88]. Parents of each gene (genes that regulate a gene are called parents of that gene) were determined using this algorithm; the parents can be protein complex or other genes. Principal component analysis was used to find the protein complexes that were involved in regulating certain genes. Three gene networks were estimated: (i) by using only microarray data (ii) by using only p-p interaction data and (iii) by using the greedy hill-climbing algorithm. The three networks were then compared with the KEGG compiled network for evaluation. The network edges agreed with the KEGG pathway used for comparison. By using 350 chosen genes from the MIPS functional category *mitotic cell cycle and cell cycle control*, 34 protein complexes were discovered in this study (22 of these 34 complexes are listed in MIPS complex catalog). Also, incorporating phase information of cell cycle (e.g. G1/S, S, M phase) revealed biologically important relationships of several genes that are not included in the KEGG pathway.

 It is well established that inferring GRNs from mRNA data alone has a huge computational complexity and often lack in accuracy. Thus, a number of studies [88-90] used prior biological knowledge to reduce the complexity and/or incorporated other type(s) of data to increase the accuracy of the inference process. But to our knowledge, no such study has been done that uses protein abundance data along with transcriptomic data to infer a GRN which can reveal a dynamic relationship model between transcriptomic and proteomic network.

## 6 DISCUSSION

 Studies have shown that there exists a poor correlation between mRNA expression and protein abundance [1-5]. Some possible reasons based on protein half-lives, post transcription machinery etc. has been proposed. It is interesting to locate analogies between biological scenarios and other physical scenarios so that approaches used for the analysis of one can throw insights and be possibly used for the analysis of the other. For instance, Wang *et al*. [128] compared the gene-mRNA-protein structure with computer’s internal structure and proposed a theory that can be used to verify the reason behind the poor correlations.

 On a similar note, we propose that a gene-transcriptome-proteome network has a number of similarities with an organizational command structure. We next show the relations between the two using a military command system. 

 The military headquarter can be assumed as the main data processor and command center during war time. Headquarter send commands to the base camp that actually controls the on-field troops. The on-field troops directly take part in the operation. Command and direction of the headquarter can be viewed as the gene sequence in DNA which encodes the proteins. The base camp command can be viewed as the transcriptome and the on-field troops can be viewed as the proteome. The factors that affect any on-field troop in the operation can be viewed as metabolites and other external conditions. A troop may not be always able to exactly perform as commanded and sometimes the base camp cannot pass on the exact command from the head quarter due to on-field scenario. Furthermore, the base camp may have a different strategy to implement the commands relayed from the headquarter resulting in a delay in the implementation of the headquarter command. Thus, reflection of any command may not be instantaneous. Similarly, transcriptomic existence does not guarantee instantaneous or even delayed version of proteomic abundance.

 Furthermore, a command from the headquarters may not require completion depending on the on-field situation and instantaneous thinking. Thus, delay of the command propagation plays an important role. Similarly, the delay in creating a protein from gene-mRNA-protein (central dogma) system may cause the desired protein ‘not needed’ at all.

 The above mentioned analogy can be supported by the fact that the command may be adaptively changed based on the needs of the troop or success i.e. there is a feedback from the troop which may alter the war-plan. Similarly, feedback from outside factors and proteins can control the ’on’ and ’off’ mechanism of genes. Thus, to understand the biological mechanism and associated network, we need to have a detailed idea of how the proteins and mRNAs react to outside stimulations and how the commands from the genes are neglected resulting in a poor correlation between transcriptomic and proteomic data. In brief, we need to broaden our view just from transcriptomic abundance and proteomic abundance and consider an integrated transcriptomic-proteomic approach incorporating other factors such as external conditions, metabolites etc. The relation between transcriptomic and proteomic domain can be better understood if a time series of gene and protein expression for single cells are available for a good length of time with high sampling frequency.

 As mentioned earlier, similarities between different domains allow us to apply techniques developed for one domain in another and also provide unique viewpoints for understanding the system behavior. Since biological networks are extremely complex and large-scale, a natural question arises whether we can relate them to other complex networks such as social networks, communication networks, web graphs etc. [129-131]. The recent development of online social networks offers an analogy between the molecular biology networks and social networks. The social networks can be considered to have two primary domains: one of them consists of the network of physical relations as manifested through verbal communications in workplaces, schools, neighborhood etc. and the other is the network of online relations through social media such as facebook, LinkedIn, twitter, Wikipedia, play station networks etc. We can associate the transcriptomic domain as the physical relation network and the proteomic domain as the social media network. The portion of individuals participating in social media can be considered as the protein coding genes. The social media network and the physical social networks are both connected and can have very similar communities just like related set of mRNAs and proteins are involved in specific functions. The commands of protein generation through mRNAs are similar to individuals expressing their views on social media. There will be links in physical social networks and links in virtual social networks and multitude of cross-links between these two networks. A number of views of an individual may not be instantly reflected in the social media due to surrounding physical situations, delay in reaching the device to post the message or disturbances in the online network. Expressions of emotions in the physical world are quick and can change fast similar to mRNAs that are mostly transient. Due to the vast memory of online interactions, emotion expressed in the social media remains for longer time similar to the scenario of generated proteins being in the system for longer time. Assuming this scenario, we can ask questions such as does the problem of learning the actual emotions of individuals of the social network by asking online questions equivalent to understanding the biological regulatory mechanism by studying protein abundance alone? In a social network, an individual’s bad disposition can affect the moods of closely linked people around him/her (such as members of his/her community in the social network) just like RNAs can affect other surrounding RNAs. However, the manifestation of an individual’s gloomy state of mind in the social media network may not have the same effect as observed in the physical social network whereas some other expressions in the online world can influence more links in the online and physical networks as compared to the expression propagated through the physical social network. In a similar fashion, some proteins can have much more effect on surrounding proteins under specific conditions as compared to the effect of the corresponding mRNA on its surrounding mRNAs. As a single time snapshot is not sufficient to detect how information is spreading through a social network, single time snapshots of mRNAs and Proteins are not suitable for understanding the biological machinery. Time series data of mRNAs and Proteins for individual cells are required to get better understanding of the interactions of the transcriptomic and proteomic domains. 

## 7 CONCLUSIONS

 As compared to existing reviews [8, 7, 6, 10] on joint transcriptomic and proteomic profiling, the current article focuses on uncovering the primary categories of approaches that have been proposed for fusion of transcriptomic and proteomic data. We have divided the existing methods into eight main categories and illustrated each by specific example of studies. For a researcher searching for ways to combine a set of transcriptomic and proteomic profiles, this review provides a concise overview of the existing analysis techniques categorized into eight types and the advantages and limitations of the various approaches. For further insights, we provide analogies of the transcriptomic and proteomic expression scenario with cases in large scale organizational and social networks. This can possibly allow design of methodologies for joint analysis of mRNA and Protein expression data based on fusion techniques applied in other large scale network analysis.

## Figures and Tables

**Fig. (1):**
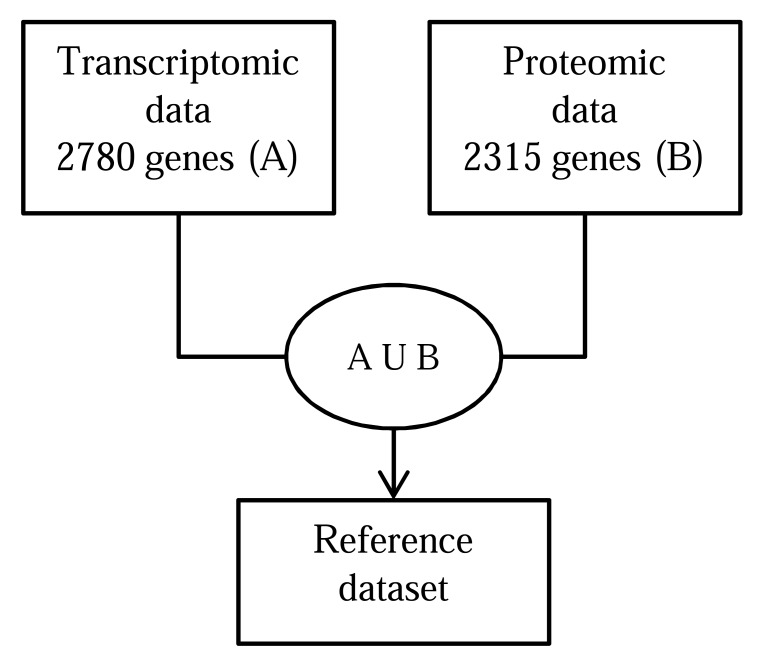
Integration of transcriptomic and proteomic dataset by simple union method.

**Fig. (2):**
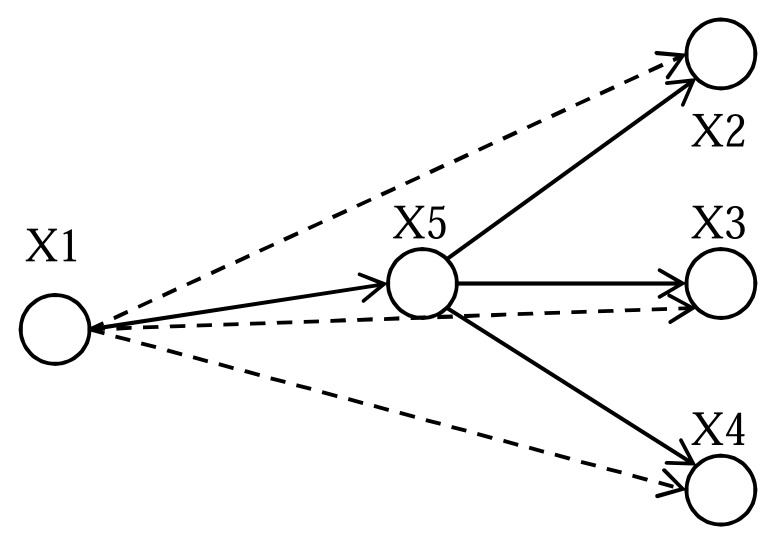
Hidden node analysis reveals new node *X*5. The dotted line represents the connectivity before hidden node analysis and the solid line reresents the connectivity after hidden node analysis.

**Fig. (3):**
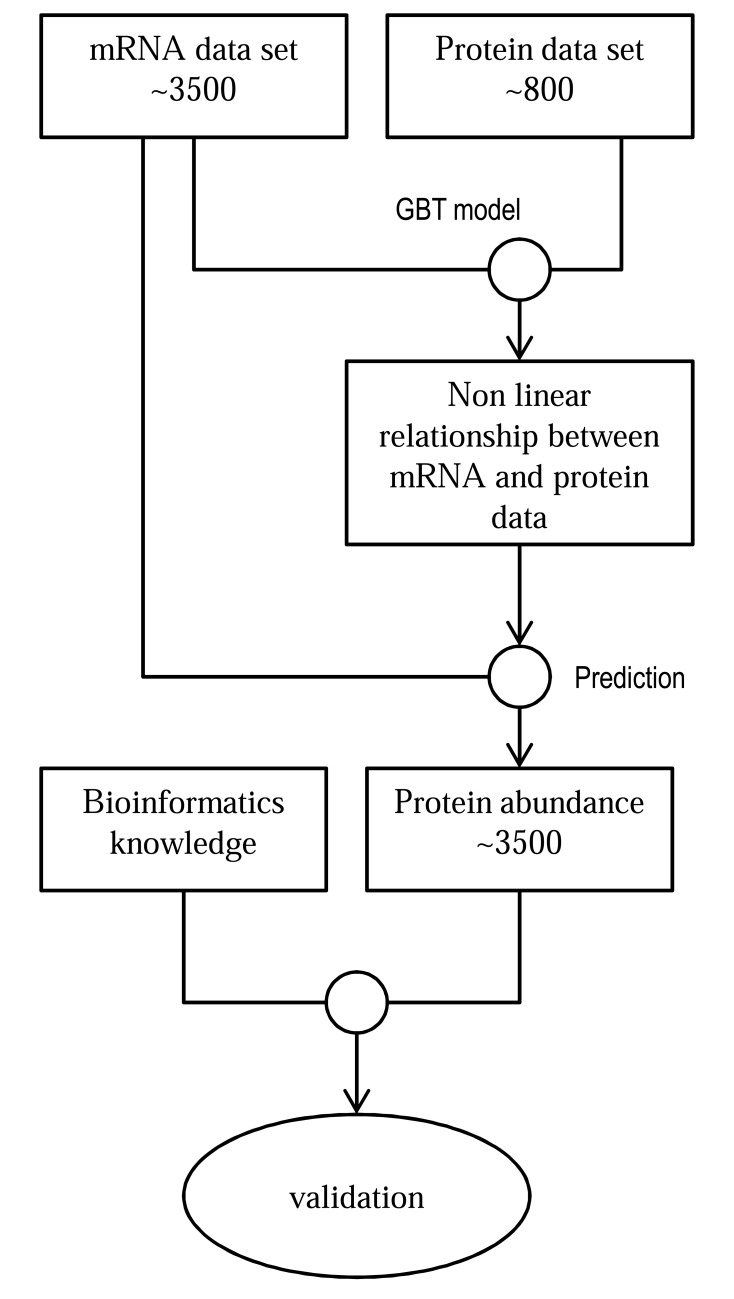
Flowchart of the method used by Garcia et al. [77].

**Fig. (4):**
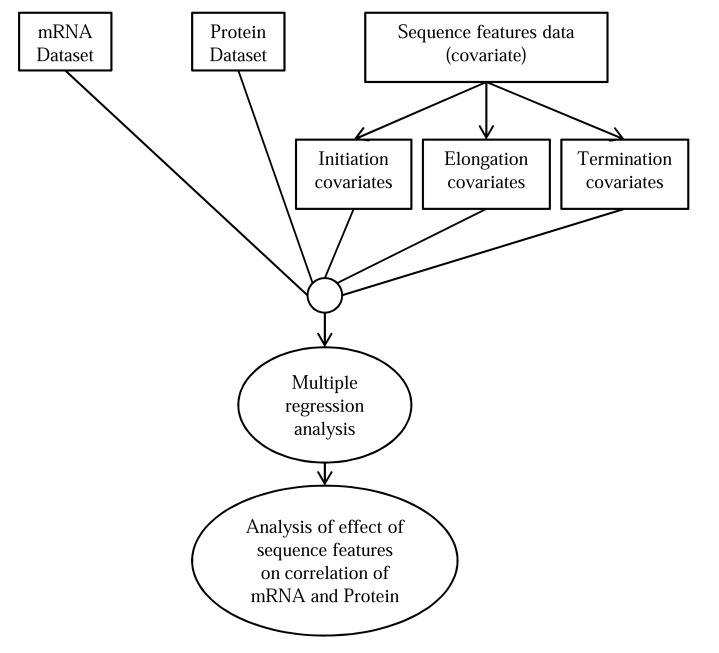
Finding effect of sequence features on mRNA-protein correlation by multiple regression analysis.

**Fig. (5):**
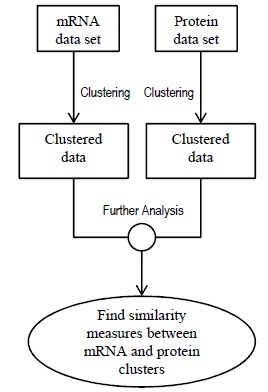
Method for clustering individually.

**Fig. (6):**
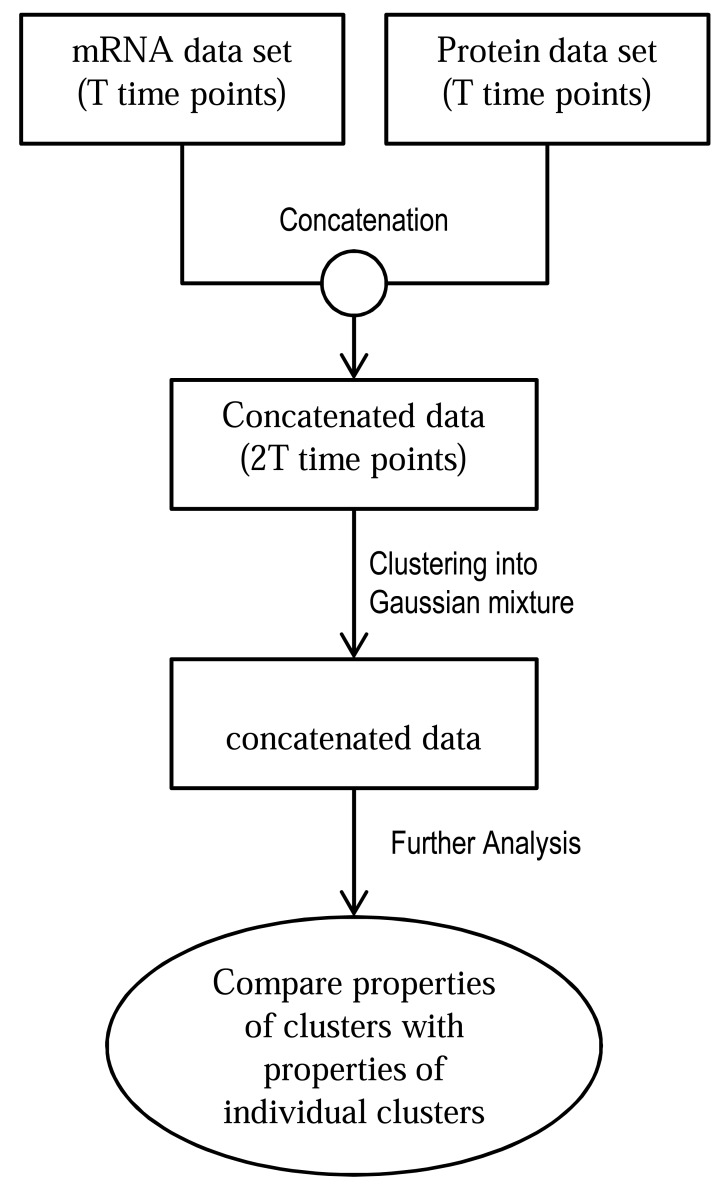
Method for clustering after concatenation.

**Fig. (7):**
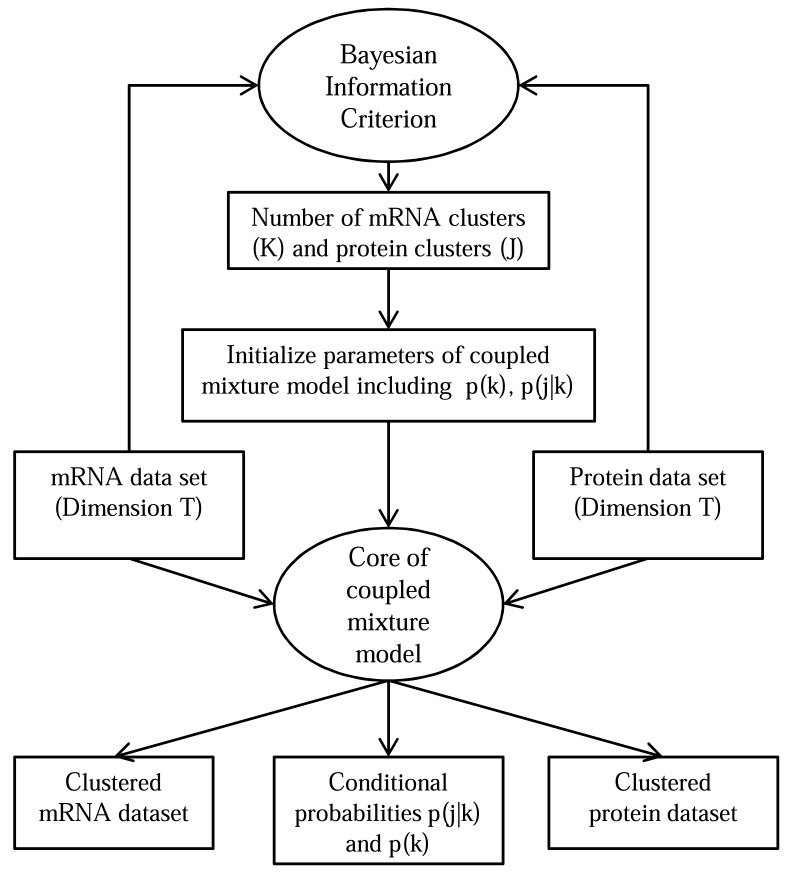
Coupled clustering method used by Rogers *et al.* [82].

**Fig. (8):**
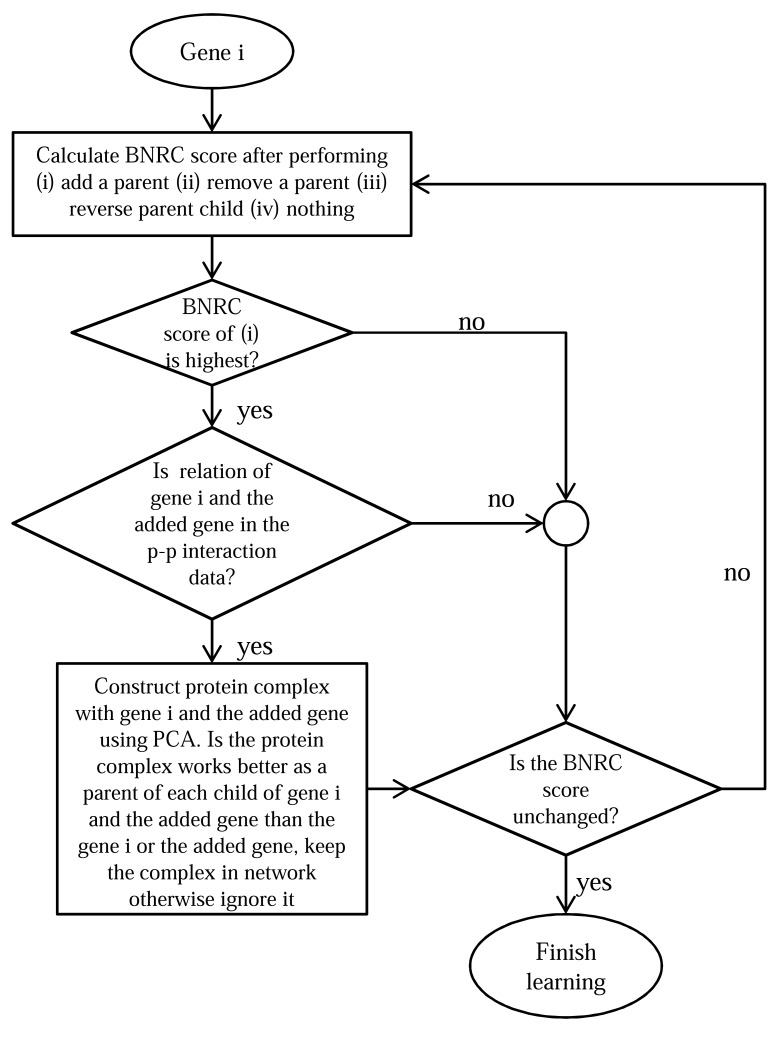
The greedy hill-climbing algorithm for finding and modeling protein complexes and estimating a gene network.

## Footnotes

1
*http://www.ncbi.nlm.nih.gov/About/primer/est.html*

2 Gene Ontology annotation tool, *http://www.geneontology.org/GO.tools_by_type.browser.shtml*

3 Ingenuity systems, USA, *http://www.ingenuity.com/*

4 details of this symbiotic relation can be found at: *http://web.mst.edu/djwesten/Bj.html*

5
*http://genome.jgi.doe.gov/soybean/soybean.info.html*

6 The 15 categories are *(i)* Amino acid biosynthesis *(ii)* Biosynthesis of cofactors, prosthetic groups and carriers, *(iii)* Cell envelope *(iv)* Cellular processes *(v)* Central intermediary metabolism *(vi)* Energy metabolism *(vii)* Fatty acid, phospholipid and sterol metabolism *(viii)* Purines, pyrimidines, nucleosides, and nucleotides *(ix)* Regulatory functions *(x)* DNA replication, recombination, and repair *(xi)* Transcription *(xii)* Translation *(xiii)* Transport and binding proteins *(xiv)* Hypothetical and *(xv)* other categories. These were found from *http://genome.kazusa.or.jp/rhizobase/Bradyrhizobium/genes/category.*

7 HUPDB v2.0 Human Urinary Proteom database *http://mosaiquesdiagnostics.de/diapatpcms/mosaiquescms/front_content.php?idcat=257*

8 These 4 genes are COL15A1, UMOD, PTGDS and APOA1

9 The 27 biological processes are: Protein metabolism and modification, Blood circulation and gas exchange, Cell structure and motility, Development processes, Immunity and defense, Protein modification, Signal truansduction, Cell structure, Cell motility, Intracellular protein traffic, Cell cycle, Cell adhession, Cell communication, Intracellular signalling cascade, Mesoderm development, Mitosis, Ectoderm development, Protein phosphorylation, Blood clotting, Cell proliferation and diferentiation, Cell cycle control, Neurogenesis, Homeostasis, Interferon-mediated immunity, Angiogenesis, Chromosome segregation, Apoptosis

10 The 7 pathways are: Cell communication, ECM-receptor interaction, p53 signaling pathway, Complement and coagulation cascades, Tight junctions, Regulation of active cytoskeleton, Focal adhesion

11
*http://mapper.chip.org/*

12
*http://www.genego.com/metacore.php*

13 Hidden node analysis: *http://www.genego.com/hidden_nodes.php*

14
*r_cucleolus_*=0.8, *r_cellperiphery_*=0.74, *r_cellcycle_*=0.71

15
*r_mitochondria_*=0.42, *r_cellrescue_*=0.45

16
*http://www.ncbi.nlm.nih.gov/IEB/ToolBox/SDKDOCS/SEQFEAT.HTML*

## References

[1] Guoan  Chen , Tarek G Gharib, Chiang-Ching  Huang , Jeremy M G Taylor, David  E Misek, Sharon  L R Kardia, Thomas  J Giordano, Gharib Chiang-Ching Huang Jeremy M G, Mark  D Iannettoni, Mark  B Orringer, Samir  M Hanas, David  G Beer (2002). "Discordant protein and mrna expression
in lung adenocarcinomas". Molecular and Cellular Proteomics.

[2] Laura E Pascal1 , Lawrence D True , Eric W Deutsch , David S Campbell , Michael  Risk, Ilsa M Coleman , Lillian J Eichner , Peter S Nelson , Alvin Y Liu (2008). “Correlation of mrna and protein levels: Cell type-specific gene expression of cluster designation antigens in the prostate". BMC Genomics.

[3] Gygi SP, Rochon Y, Franza BR, Aebersold R (1999). “Correlation between protein and mRNA abundance in yeast”. Mol Cell Biol.

[4] Edward Yeung S (2011). “Genome-wide correlation between mrna and protein in a single cell”. Angewandte Chemie Inter-national Edition.

[5] Anatole  Ghazalpour, Brian  Bennett, Vladislav  A Petyuk, Luz  Orozco, Raffi  Hagopian, Imran  N Mungrue, Charles  R Farber, Janet  Sinsheimer, Hyun  M Kang, Nicholas  Furlotte, Christopher  C Park, Ping-Zi  Wen, Heather  Brewer, Karl  Weitz, David  G Camp II, Calvin  Pan, Roumyana  Yordanova, Isaac  Neuhaus, Charles  Tilford, Nathan  Siemers, Peter  Gargalovic, Eleazar  Eskin, Todd  Kirchgessner, Desmond J Smith, Richard  D Smith, Aldons  J Lusis (2011). "Comparative analysis of proteome and transcriptome variation in mouse". PLoS Genet.

[6] Catherine Jane Hack (2004). “Integrated transcriptome and proteome data: The challenges ahead". Briefings in funtional genomics and proteomics.

[7] Brian Cox, Thomas Kislingera, Andrew Emili (2005). “Integrating gene and protein expression data: pattern analysis and profile mining”. Methods.

[8] Lei Nie, Gang Wu, David E Culley , Johannes C M Scholten , Weiwen Zhang (2007). “Integrative analysis of tran-scriptomic and proteomic data: challenges, solutions and applications”. Critical Reviews In Biotechnology.

[9] Michael Hecker, Sandro Lambeck, Susanne Toepfer, Eugene van Someren, Reinhard Guthke (2009). “Gene regula-tory network inference: Data integration in dynamic models—a review”. Biosystems.

[10] Simon Rogers (2011). “Statistical methods and models for bridging omics data levels”. Methods in Molecular Biology.

[11] Michael Heller J (2002). “Dna microarray technology: Devices, systems, and applications". Annual Review of Biomedical Engineering.

[12] Ed M Southern (2000). “Blotting at 25”. Trends in Biochemical Sciences.

[13] Pieter Vos, Rene Hogers, Marjo Bleeker, Martin Reijans, Theo van de Lee, Miranda Hornes, Adrie Friters, Jerina Pot, Johan Paleman, Martin Kuiper, Marc Zabeau (1995). “Aflp: a new technique for dna fingerprinting”. Nucleic Acids Research.

[14] Punchapat Sojikul, Panida Kongsawadworakul, Unchera Viboonjun, Jittrawan Thaiprasit, Burapat Intawong, Jarunya Narangajavana, Mom Rajawong Jisnuson Svasti (2010). “Aflp-based transcript profiling for cassava genome-wide expression analysis in the onset of storage root formation”. Physiologia Plantarum.

[15] Michel Claverie, Marlène Souquet, Janine Jean, Nelly Forestier-Chiron, Vincent Lepitre, Martial Pr , John Jacobs, Danny Llewellyn, Jean-Marc Lacape (2012). “cdna-aflp-based genetical genomics in cotton fibers”. TAG The-oretical and Applied Genetics.

[16] Ge Xiaomeng, Chen Weihua, Song Shuhui, Wang Weiwei, Hu Songnian, Yu Jun (2008). “Transcriptomic profiling of mature embryo from an elite super-hybrid rice lyp9 and its parental lines”. BMC Plant Biology.

[17] MD  Adams, JM  Kelley, JD  Gocayne, M  Dubnick, MH  Polymeropoulos, H  Xiao, CR  Merril,  A  Wu, B  Olde, RF  Moreno (1991). “Complementary dna sequencing: expressed sequence tags and human genome project”. Science.

[18] Victor E Velculescu, Lin Zhang , Bert Vogelstein, Kenneth W Kinzler (1995). “Serial analysis of gene expression”. Science.

[19] Colleen D Hough , Cheryl A Sherman-Baust,  Ellen S Pizer, F  J Montz , Dwight D Im , Neil B Rosenshein , Kathleen R Cho , Gregory J Riggins , Patrice J Morin (2000). “Large-scale serial analysis of gene expression reveals genes differentially expressed in ovarian cancer”. Cancer Researc.

[20] S  Brenner, M  Johnson, J  Bridgham, G  Golda, DH  Lloyd,  D  Johnson, S  Luo, S  McCurdy, M  Foy, M  Ewan, R  Roth, D  George, S  Eletr, G  Albrecht, E  Vermaas, S R Williams, K  Moon, T  Burcham, M  Pallas, RB  DuBridge, J  Kirchner, K  Fearon,  J Mao, K  Corcoran (2000). “Gene expression analysis by massively parallel signature sequencing (mpss) on microbead arrays”. Nature Biotechnology.

[21] Deana Erdner, Donald Anderson (2006). “Global transcriptional profiling of the toxic dinoflagellate alexandrium fundyense using massively parallel signature sequencing”. BMC Genomics.

[22] Rachael Natrajan, Alan Mackay, Maryou B Lambros, Britta Weigelt, Paul M Wilkerson, Elodie Manie, Anita Grigoriadis, Roger A'Hern, Petra van der Groep, Iwanka Kozarewa, Tatiana Popova, Odette Mariani, Samra Turajlic, Simon J Furney, Richard Marais, Daniel-Nava Rodruigues, Adriana C Flora, Patty Wai, Vidya Pawar, Simon McDade, Jason Carroll, Dominique Stoppa-Lyonnet, Andrew R Green, Ian O Ellis, Charles Swanton, Paul van Diest, Olivier Delattre, Christopher J Lord, William D Foulkes, Anne Vincent-Salomon, Alan Ashworth, Marc Henri Stern, Jorge S Reis-Filho (2012). “A whole-genome massively parallel sequencing analysis of brca1 mutant oestrogen receptor-negative and -positive breast cancers”. The Journal of Pathology.

[23] Zhong Wang, Mark Gerstein, Michael Snyder (2009). “Rna-seq: a revolutionary tool for transcriptomics”. Nature Reviews. Genetics.

[24] Ugrappa Nagalakshmi, Zhong Wang, Karl Waern, Chong Shou, Debasish Raha, Mark Gerstein, Michael Snyder (2008). “The transcriptional landscape of the yeast genome defined by rna sequencing”. Science.

[25] Brian T Wilhelm, Samuel Marguerat, Stephen Watt, Falk Schubert, Valerie Wood, Ian Goodhead, Christopher J Penkett, Jane Rogers, Jürg B hler (2008). “Dynamic repertoire of a eukaryotic transcriptome surveyed at single-nucleotide resolution”. Nature.

[26] Ali Mortazavi, Brian A Williams, Kenneth McCue, Lorian Schaeffer, Barbara Wold (2008). “Mapping and quantifying mammalian transcriptomes by rna-seq”. Nature Methods.

[27] Ryan Lister, Ronan C O'Malley, Julian Tonti-Filippini, Brian D Gregory , Charles C Berry, A Harvey Millar, Joseph R Ecker (2008). “Highly integrated single-base resolution maps of the epigenome in Arabidopsis”. Cell.

[28] Nicole Cloonan, Alistair R R Forrest, Gabriel Kolle, Brooke B A Gardiner, Geoffrey J Faulkner, Mellissa K Brown, Darrin F Taylor, Anita L Steptoe, Shivangi Wani, Graeme Bethel, Alan J Robertson , Andrew C Perkins , Stephen J Bruce , Clarence C Lee , Swati S Ranade , Heather E Peckham , Jonathan M Manning , Kevin J McKernan , Sean M Grimmond (2008). “Stem cell transcriptome profiling via massive-scale mrna sequencing”. Nature Methods.

[29] John C Marioni , Christopher E Mason , Shrikant M Mane , Matthew Stephens, Yoav Gilad (2008). “Rna-seq: An assessment of technical reproducibility and comparison with gene expression arrays”. Genome Research.

[30] Nathan Blow (2009). “Transcriptomics: The digital generation". Nature.

[31] Nicole C Roy, Eric Altermann , Zaneta A Park , Warren C McNabb (2011). “A comparison of analog and next-generation transcriptomic tools for mammalian studies”. Briefings in Functional Genomics.

[32] Kristin Schirmer, Beat B Fischer , Danielle J Madureira , Smitha Pillai (2010). “Transcriptomics in ecotoxicology”. Analytical and Bioanalytical Chemistry.

[33] Henriques A, Gonzalez De Aguilar JL (2011). “Can transcriptomics cut the gordian knot of amyotrophic lateral sclerosis?”. Current Genomics.

[34] Zhan Zhou, Jianying Gu, Yi-Ling Du, Yong-Quan Li, Yufeng Wang (2011). “The -omics era- toward a systems-level understanding of streptomyces”. Current Genomics.

[35] S Michael Rothenberg, Jeff Settleman (2010). “Discovering tumor suppressor genes through genome-wide copy number analysis”. Current Genomics.

[36] Thierry Rabilloud, Cécile Lelong (2011). “Two-dimensional gel electrophoresis in proteomics: A tutorial". Journal of Proteomics.

[37] Eleonora Piruzian, Sergey Bruskin, Alex Ishkin, Rustam Abdeev, Sergey Moshkovskii, Stanislav Melnik, Yuri Nikolsky, Tatiana Nikolskaya (2010). “emphIntegrated network analysis of transriptomic and proteomic data in psoriasis”. BMC Systems Biology.

[38] Dov Greenbaum, Ronald Jansen, Mark Gerstein (2002). “Analysis of mRNA expression and protein abundance data: an approach for the comparison of the enrichment of features in the cellular population of proteins and transcrips”. Bioinformatics.

[39] Emmanuelle Com, Eric Boitier, Jean-Pierre Marchandeau, Arnd Brandenburg, Susanne Schroeder, Dana Hoffmann, Angela Mally, Jean-Charles Gautier (2012). “Integrated transcriptomic and proteomic evaluation of gentamicin nephrotoxicity in rats”. Toxicology and Applied Pharmacology.

[40] Jun X Yan , Angelica T Devenish , Robin Wait, Tim Stone, Steve Lewis, Sue Fowler (2002). “Fluorescence two-dimensional difference gel electrophoresis and mass spectrometry based proteomic analysis of escherichia coli”. PROTEOMICS.

[41] Rita Marouga, Stephen David, Edward Hawkins (2005). “The development of the dige system: 2d fluorescence difference gel analysis technology". Analytical and Bioanalytical Chemistry.

[42] Julien Franck, Karim Arafah, Mohamed Elayed, David Bonnel, Daniele Vergara, Amélie Jacquet, Denis Vinatier, Maxence Wisztorski, Robert Day, Isabelle Fournier, Michel Salzet (2009). “Maldi imaging mass spectrometry”. Molecular and Cellular Proteomic.

[43] M  Reid Groseclose, Pierre P Massion, Pierre Chaurand, Richard M Caprioli (2008). “High-throughput proteomic analysis of formalin-fixed paraffin-embedded tissue microarrays using maldi imaging mass spectrometry”. PROTEOMICS.

[44] Mareike Elsner, Sandra Rauser, Stefan Maier, Cédrik Sch ne, Benjamin Balluff, Stephan Meding, Gerhard Jung, Martin Nipp, Hakan Sarioglu, Giuseppina Maccarrone, Michaela Aichler, Annette Feuchtinger, Rupert Langer, Uta Jütting, Marcus Feith, Bernhard Küster, Marius Ueffing, Horst Zitzelsberger, Heinz Höfler, Axel Walch (2012). “Maldi imaging mass spectrometry reveals cox7a2, tagln2 and s100-a10 as novel prognostic markers in barrett’s adenocarcinoma”. Journal of Proteomics.

[45] Guihua Yue, Quanzhou Luo, Jian Zhang, Shiaw-Lin Wu, Barry  L Karger (2007). “Ultratrace lc/ms proteomic analysis using 10-Î¼m-i porous layer open tubular poly(styrene−divinylbenzene) capillary columns". Analytical Chemistry.

[46] Dwayne A Elias , Matthew E Monroe , Matthew J Marshall , Margaret F Romine , Alexander S Belieav , James K Fredrickson , Gordon A Anderson , Richard D Smith , Mary Lipton S (2005). “Global detection and characterization of hypothetical proteins in shewanella oneidensis mr-1 using lc-ms based proteomics”. PROTEOMICS.

[47] Lei Nie, Gang Wu, Fred J Brockman, Weiwen  Zhang (2006). “Integrated analysis of transcriptomic and proteomic data of desulfovibrio vulgaris: zero-inflated poisson regression models to predict abundance of undetected proteins”. Bioinformatics.

[48] Ravindra Varma Polisetty, Poonam Gautam, Rakesh Sharma, H C Harsha, Sudha C Nair, Manoj >Kumar Gupta, Megha S Uppin , Sundaram Challa, Aneel Kumar Puligopu, Praveen Ankathi, Aniruddh K Purohit, Giriraj R Chandak , Akhilesh Pandey, Ravi Sirdeshmukh (2012). “Lc-ms/ms analysis of differentially expressed glioblastoma membrane proteome reveals altered calcium signalling and other protein groups of regulatory functions”. Molecular and Cellular Proteomics.

[49] Nathanael Delmotte, Christian H Ahrens, Claudia Knief, Ermir Qeli, Marion Koch, Hans-Martin Fischer , Julia A Vorholt, Hauke  Hennecke, Gabriella Pessi (2010). “An integrated proteomics and transcriptomics reference data set provides new insights into the Bradyrhizobium japonicum bacteroid metabolism in soybean root nodules”. Proteomics.

[50] Lau Sennels, Mogjiborahman Salek, Lee Lomas, Egisto Boschetti, Pier Giorgio Righetti, Juri Rappsilber (2007). “Pro-teomic analysis of human blood serum using peptide library beads”. Journal of Proteome Research.

[51] Leann M Mikesh, Beatrix Ueberheide, An Chi , Joshua J Coon , John EP, Syka Jeffrey Shabanowitz, Donald F Hunt  (2006). “The utility of etd mass spectrometry in proteomic analysis”. Biochimica et Biophysica Acta (BBA) - Proteins and; Proteomics.

[52] Henrik Molina, David M Horn, Ning Tang , Suresh Mathivanan, Akhilesh Pandey (2007). “Global proteomic profiling of phosphopeptides using electron transfer dissociation tandem mass spectrometry”. Proceedings of the National Academy of Sciences.

[53] Danielle L Swaney , Craig D Wenger , James A Thomson, Joshua J Coon (2009). “Human embryonic stem cell phosphoproteome revealed by electron transfer dissociation tandem mass spectrometry”. Proceedings of the National Academy of Sciences.

[54] Brett Spurrier, Sundhar Ramalingam, Satoshi Nishizuka (2008). “Reverse-phase protein lysate microarrays for cell signaling analysis”. Nat. Protocols.

[55] Satoshi Nishizuka, Lu Charboneau, Lynn Young, Sylvia Major, William C Reinhold, Mark  Waltham, Hosein Kouros-Mehr, Kimberly J Bussey, Jae  K Lee, Virginia  Espina, Peter J Munson, Emanuel  Petricoin, Lance A Liotta, John N Weinstein  (2003). “Proteomic profiling of the nci-60 cancer cell lines using new high-density reverse-phase lysate microarrays”. Proceedings of the National Academy of Sciences.

[56] Stacy M Cowherd , Virginia A Espina , Emanuel F Petricoin, Lance  A Liotta (2004). “Proteomic analysis of human breast cancer tissue with laser-capture microdissection and reverse-phase protein microarrays”. Clinical Breast Cancer.

[57] Y  Baskin, T  Yigitbasi (2010). “Clinical proteomics of breast cancer”. Current Genomics.

[58] Shine J, Dalgarno L (1974). “The 3’-terminal sequence of escherichia coli 16s ribosomal rna: complementarity to nonsense triplets and ribosome binding sites”. Proc Natl Acad Sci U S A.

[59] Shine J, Dalgarno L (1975). “Determinant of cistron specificity in bacterial ribosomes”. Nature.

[60] Alistair HA Bingham, Sreenivasan Ponnambalam , Bernard Chan, Stephen Busby (1986). “Mutations that reduce expression from the p2 promoter of the escherichia coli galactose operon”. Gene.

[61] Grossman AD, Zhou YN, Gross C, Heilig J, Christie GE, Calendar R (1985). “Mutations in the rpoh (htpr) gene of escherichia coli k-12 phenotypically suppress a temperature-sensitive mutant defective in the sigma 70 subunit of rna polymerase”. J Bacteriol.

[62] Claes Gustafsson, Sridhar Govindarajan, Jeremy Minshull (2004). “Codon bias and heterologous protein expression”. Trends in Biotechnology.

[63] Paul M Sharp ,  Wen-Hsiung Li (1987). “The codon adaptation index-a measure of directional synonymous codon usage bias, and its potential applications”. Nucleic Acids Research.

[64] Gila Lithwick, Hanah Margalit (2003). “Hierarchy of sequence-dependent features associated with prokaryotic translation”. Genome Research.

[65] Eldad N, Arava Y (2008). “A ribosomal density-mapping procedure to explore ribosome positions along translating mrnas”. Methods Mol Biol.

[66] Nicholas T Ingolia , Sina Ghaemmaghami, John R S Newman, Jonathan  S Weissman (2009). “Genome-wide analysis *in vivo* of translation with nucleotide resolution using ribosome profiling”. Science.

[67] Dov Greenbaum, Christopher Colangelo, Kenneth Williams, Mark Gerstein (2003). “Comparing protein abundance and mRNA expression levels on a genomic scale”. Genome Biology.

[68] Raymond J Cho , Michael J Campbell , Elizabeth A Winzeler , Lars Steinmetz, Andrew  Conway , Lisa  Wodicka , Tyra G  Wolfsberg , Andrei E Gabrielian , David Landsman, David J Lockhart , Ronald W Davis (1998). “A genome-wide transcriptional analysis of the mitotic cell cycle”. Molecular cell.

[69] James L Hargrove, Frederick Schmidt H (1989). “The role of mRNA and protein stability in gene expression”. The FASEB Journal.

[70] Bjorn Schwanhausser, Dorothea Busse, Na Li, Gunnar Dittmar, Johannes Schuchhardt, Jana Wolf, Wei Chen, Matthias Selbach (2011). “Global quantification of mammalian gene expression control”. Nature.

[71] Andreas Bachmair, Daniel Finley, Alexander Varshavsky (1986). “In vivo half-life of a protein is a function of its amino-terminal residue” Science.

[72] Tobias Maier, Marc Güell, Luis Serrano (2009). “Correlation of mrna and protein in complex biological samples”. FEBS Letters.

[73] Susan B Altenbach , William H Vensel , Frances M DuPont (2010). “Integration of transcriptomic and proteomic data from a single wheat cultivar provides new tools for understanding the roles of individual alpha gliadin proteins in flour quality and celiac disease”. Journal of Cereal Science.

[74] J P McRedmond , S D Park , D F Reilly, J A Coppinger , P B Maguire , D C Shields, D J Fitzgerald (2004). “Inte-gration of proteomics and genomics in platelets”. Molecular and Cellular Proteomics.

[75] Paul Perco, Irmgard Muhlberger, Gert Mayer, Rainer Oberbauer, Arno Lukas, Bernd Mayer (2010). “Linking tran-scriptomic and proteomic data on the level of protein interaction network”. Electrophoresis.

[76] Marcin Imielinski, Sangwon Cha, Tomas Rejtar, Elizabeth A Richardson, Barry L Karger , Dennis C Sgroi (2012). “Integrated proteomic, transcriptomic, and biological network analysis of breast carcinoma reveals molecular features of tumorigenesis and clinical relapse”. Molecular and Cellular Proteomics.

[77] Wandaliz Torres-García, Weiwen Zhang, George C Runger , Roger H Johnson, Deirdre  R Meldrum (2009). “Integrative analysis of transcriptomic and proteomic data of desulfovibrio vulgaris: a non-linear model to predict abundance of undetected proteins”. Bioinformatics.

[78] Jerome  H Friedman (2002). “Stochastic gradient boosting”. Computational Statistics and Data Analysis.

[79] Wandaliz Torres-Garcia, Steven D Brown , Roger H Johnson , Weiwen Zhang, George C Runger, Deirdre  R Meldrum (2011). “Integrative analysis of transcriptomic and proteomic data of shewanella oneidensis: missing value imputation using temporal datasets”. Mol. BioSyst.

[80] Feng Li, Lei Nie, Gang Wu, Jianjun Qiao, Weiwen Zhang (2011). “Prediction and characterization of missing proteomic data in desulfovibrio vulgaris”. Comparative and Functional Genomics.

[81] Lei Nie, Gang Wu, Weiwen Zhang (2006). “Correlation of mRNA Expression and Protein Abundance Affected by Multiple Sequence Features Related to Translational Efficiency in Desulfovibrio vulgaris: A Quantitative Analysis". Genetics Society of America.

[82] Simon Rogers, Mark Girolami, Walter Kolch, Katrina M Waters, Tao Liu, Brian Thrall, H Steven Wiley (2008). “Investigating the correspondence between transcriptomic and proteomic expression profiles using coupled cluster models”. Bioinformatics.

[83] S  Liang, S  Fuhrman, R  Somogyi (1998). “Reveal, a general reverse engineering algorithm for inference of genetic network architectures”. Pacific Symposium on Biocomputing.

[84] P  D’haeseleer (1999). “Linear modeling of mrna expression levels during cns development and injury”. Pacific Symposium on Biocomputing.

[85] Reinhard Guthke, Ulrich Möller, Martin Hoffmann, Frank Thies, Susanne Töpfer (2005). “Dynamic network reconstruction from gene expression data applied to immune response during bacterial infection”. Bioinformatics.

[86] Edward R Dougherty (2007). “Validation of inference procedures for gene regulatory networks”. Current Genomics.

[87] N  Friedman, M  Linial, I  Nachman, D  Pe’er (2000). “Bayesian networks to analyze expression data”. Proceedings of the Fourth Annual International Conference on Computational Molecular Biology.

[88] N  Nariai, S  Kim, S  Imoto, S  Miyano (2004). “Using protein-protein interactions for refining gene networks estimated from microarray data by bayesian networks”. Pacific Symposium on Biocomputing.

[89] Yu Zhang, Zhidong  Deng , Hongshan Jiang, Peifa Jia (2007). “Inferring gene regulatory networks from multiple data sources via a dynamic bayesian network with structural em”. Data Integration in the Life Sciences.

[90] Adriano V Werhli, Dirk  Husmeier (2007). “Reconstructing gene regulatory networks with bayesian networks by combining expression data with multiple sources of prior knowledge”. Statistical Applications in Genetics and Molecular Biology.

[91] E  Segal, H  Wang, D  Koller (2003). “Discovering molecular pathways from protein interaction and gene expression data”. Bioinformatics.

[92] Takakazu Kaneko, Yasukazu Nakamura, Shusei Sato, Kiwamu Minamisawa, Toshiki Uchiumi, Shigemi Sasamoto, Akiko Watanabe, Kumi Idesawa, Mayumi Iriguchi, Kumiko Kawashima, Mitsuyo Kohara, Midori Matsumoto, Sayaka Shimpo, Hisae Tsuruoka, Tsuyuko Wada, Manabu Yamada, Satoshi Tabata (2002). “Complete genomic sequence of nitrogen-fixing symbiotic bacterium bradyrhizobium japonicum usda110”. DNA Research.

[93] David N Perkins, Darryl J C Pappin, David M Creasy, John S Cottrell (1999). “Probability-based protein identification by searching sequence databases using mass spectrometry data”. ELECTROPHORESIS.

[94] G  Pessi, C H Ahrens, H  Rehrauer, A  Lindemann (2007). “Genome-wide transcript analysis of Bradyrhizobium japonicum bacteroids: in soybean root nodules”. Mol. Plant Microb Interact.

[95] Annamraju D Sarma, David  W Emerich (2005). “Global protein expression pattern of bradyrhizobium japonicum bacteroids: A prelude to functional proteomics". PROTEOMICS.

[96] Schmid H, Boucherot A, Yasuda Y, Henger A, Brunner B, Eichinger F, Nitsche A, Kiss E, Bleich M, Gröne HJ, Nelson PJ, Schlöndorff D, Cohen CD, Kretzler M (2006). “Modular activation of nuclear factor-kappab transcriptional programs in human diabetic nephropathy”. Diabetes.

[97] Hans J Baelde, Michael  Eikmans , Peter  P Doran, David WP Lappin, Emile  de Heer, Jan A (2004). Bruijn, “Gene expression profiling in glomeruli from human kidneys with diabetic nephropathy”. American Journal of Kidney Dis-eases.

[98] M  Rudnicki, S  Eder, P  Perco, J  Enrich, K  Scheiber, C  Koppelstatter, G  Schratzberger, B  Mayer, R  Oberbauer, T W Meyer, G  Mayer (2006). “Gene expression profiles of human proximal tubular epithelial cells in proteinuric nephropathies”. Kidney Int.

[99] Amos Bairoch, Brigitte Boeckmann, Serenella Ferro, Elisabeth Gasteiger (2004). “Swiss-prot: Juggling between evolution and stability”. Briefings in Bioinformatics.

[100] Paul D Thomas, Anish  Kejariwal , Michael  J Campbell, Huaiyu Mi, Karen Diemer, Nan Guo, Istvan Ladunga, Betty Ulitsky-Lazareva, Anushya Muruganujan, Steven Rabkin, Jody A Vandergriff , Olivier Doremieux (2003). “Panther a browsable database of gene products organized by biological function, using curated protein family and subfamily classification”. Nucleic Acids Research.

[101] Huaiyu Mi, Qing Dong, Anushya Muruganujan, Pascale Gaudet, Suzanna Lewis, Paul  D Thomas (2010). “Panther version 7: improved phylogenetic trees, orthologs and collaboration with the gene ontology consortium”. Nucleic Acids Research.

[102] Da Wei Huang, Brad T Sherman, Richard  A Lempicki (2008). “Systematic and integrative analysis of large gene lists using david bioinformatics resources”. Nature Protocols.

[103] Andreas Bernthaler, Irmgard Muhlberger, Raul Fechete, Paul Perco, Arno Lukas, Bernd Mayer (2009). “A dependency graph approach for the analysis of differential gene expression profiles”. Mol. BioSyst.

[104] Voichita D Marinescu, Isaac  S Kohane, Alberto Riva (2005). “The mapper database: a multi-genome catalog of putative transcription factor binding sites”. Nucleic Acids Research.

[105] Yuri Nikolsky, Evgeny Sviridov, Jun Yao, Damir Dosymbekov, Vadim Ustyansky, Valery Kaznacheev, Zoltan Dezso, Laura Mulvey, Laura E Macconaill, Wendy  Winckler , Tatiana  Serebryiskaya , Tatiana  Nikolskaya , Kornelia  Polyak (2008). “Genome-wide functional synergy between amplified and mutated genes in human breast cancer”. Cancer Research.

[106] Zoltan Dezso, Yuri Nikolsky, Tatiana Nikolskaya, Jeremy Miller, David Cherba, Craig Webb, Andrej Bugrim (2009). “Identifying disease-specific genes based on their topological significance in protein networks”. BMC Syst Biol.

[107] Vasyl Pihur, Susmita Datta, Somnath Datta (2009). “Rankaggreg, an r package for weighted rank aggregation”. BMC Bioin-formatics.

[108] RY  Rubinstein, DP  Kroese (2004). The Cross-Entropy Method: A
Unified Approach to Combinatorial Optimization, Monte-Carlo
Simulation and Machine Learning.

[109] Holstege FC, Jennings EG, Wyrick JJ, Lee TI, Hengartner CJ, Green MR, Golub TR, Lander ES, Young RA (1998). “Dissecting the regulatory circuitry of a eukaryotic genome”. Cell.

[110] Frederick P Roth, Jason  D Hughes, Preston W Estep, George  M Church (1998). “Finding dna regulatory motifs within unaligned noncoding sequences clustered by whole-genome mrna quantitation”. Nat Biotech.

[111] Scott A Jelinsky, Leona  D Samson (1999). “Global response of saccharomyces cerevisiae to an alkylating agent”. Proceedings of the National Academy of Sciences.

[112] Velculescu VE, Zhang L, Zhou W, Vogelstein J, Basrai MA, Bassett DE, Hieter P, Vogelstein B, Kinzler KW (1997). “Characterization of the yeast transcriptome”. Cell.

[113] Futcher B, Latter GI, Monardo P, McLaughlin CS, Garrels JI (1999). “A sampling of the yeast proteome”. Mol Cell Biol.

[114] Washburn MP, Wolters D, Yates JR (2001). “Large-scale analysis of the yeast proteome by multidimensional protein identification technology”. Nat Biotechnol.

[115] Peng J, Elias JE, Thoreen CC, Licklider LJ, Gygi SP (2003). “Evaluation of multidimensional chromatography coupled with tandem mass spectrometry (LC/LC-MS/MS) for large-scale protein analysis the yeast proteome”. J Proteome Res.

[116] Jerome  H Friedman (2001). “Greedy function approximation: A gradient boosting machine". The Annals of Statistics.

[117] John F Heidelberg, Rekha   Seshadri , Shelley  A Haveman, Christopher L Hemme, Ian  T Paulsen, James F Kolonay, Jonathan  A Eisen, Naomi Ward, Barbara Methe, Lauren M Brinkac, Sean  C Daugherty, Robert T Deboy, Dodson, Robert J, A Scott Durkin, Ramana  Madupu, William  C Nelson, Steven A Sullivan, Derrick  Fouts ,  Daniel  H Haft, Jeremy Selengut, Jeremy D Peterson, Tanja M Davidsen, Nikhat Zafar, Liwei Zhou, Diana Radune, George Dimitrov, Mark Hance, Kevin Tran, Hoda Khouri, John Gill, Terry R Utterback, Tamara  V Feldblyum, Judy D Wall,  Gerrit  Voordouw , Claire  M Fraser (2004). “The genome sequence of the anaerobic, sulfate-reducing bacterium desulfovibrio vulgaris hildenborough”. Nat Biotech.

[118] Lei Nie, Gang Wu, Weiwen Zhang (2006). “Correlation between mrna and protein abundance in desulfovibrio vulgaris: A multiple regression to identify sources of variations". Biochemical and Biophysical Research Communications.

[119] Richard G Lomax (1992). Statistical Concepts: A Second Course for Education and the Behavioral Sciencess.

[120] Bruce  G Lindsay (1995). “Mixture models: Theory, geometry and applications”. NSF-CBMS Regional Conference Series in Probability and Statistics.

[121] A P Dempster, N M Laird, D B Rubin (1977). “Maximum likelihood from incomplete data via the em algorithm,”. Journal of the Royal Statistical Society Series B (Methodological).

[122] Marina Meila (2007). “Comparing clusterings—an information based distance”. Journal of Multivariate Analysis.

[123] Gideon Schwarz (1978). “Estimating the dimension of a model”. The Annals of Statistics.

[124] Darya Chudova, Christopher Hart, Eric Mjolsness, Padhraic Smyth (2004). “Gene expression clustering with functional mixture models”. Advances in Neural Information Processing Systems.

[125] Yihui Luan, Hongzhe Li (2003). “Clustering of time-course gene expression data using a mixed-effects model with b-splines”. Bioinformatics.

[126] Paul T Spellman, Gavin Sherlock, Michael  Q Zhang, Vishwanath R Iyer, Kirk   Anders , Michael  B Eisen, Patrick O Brown, David Botstein, Bruce Futcher (1998). “Comprehensive identification of cell cycle-regulated genes of the yeast saccharomyces cerevisiae by microarray hybridization”. Molecular Biology of the Cell.

[127] H W Mewes, D  Frishman, U  Güldener,  G  Mannhaupt, K  Mayer, M  Mokrejs, B  Morgenstern, M  Münsterkötter, S  Rudd, B  Weil (2002). “Mips: a database for genomes and protein sequences”. Nucleic Acids Research.

[128] Degeng Wang (2008). “Discrepancy between mrna and protein abundance: Insight from information retrieval process in computers". Computational Biology and Chemistry.

[129] Alain Barrat, Marc Barthlemy, Alessandro Vespignani (2008). Dynamical Processes on Complex Networks.

[130] Yong-Yeol Ahn, James P Bagrow, Sune  Lehmann (2010). “Link communities reveal multiscale complexity in networks”. Nature.

[131] Andrea Lancichinetti, Mikko Kivelä , Jari Saramäki, Santo Fortunato (2010). “Characterizing the community structure of complex networks”. PLOSOne.

